# Supplementary material for: Atorvastatin Inhibits the HIF1α-PPAR Axis, Which Is Essential for Maintaining the Function of Human Induced Pluripotent Stem Cells
Source: Mol Ther. 2018 Jun 19;26(7):1715–34. doi: 10.1016/j.ymthe.2018.06.005 (PMC6036234; doi:10.1016/j.ymthe.2018.06.005)
Supplement: Document S1. Figures S1–S8 [file mmc1.pdf]

YMTHE, Volume *26*

Supplemental Information

Atorvastatin Inhibits the HIF1α-PPAR Axis, Which Is Essential for Maintaining the Function of Human Induced Pluripotent Stem Cells

Yoshiki Nakashima, Chika Miyagi-Shiohira, Hirofumi Noguchi, and Takeshi Omasa

Figure S1

a

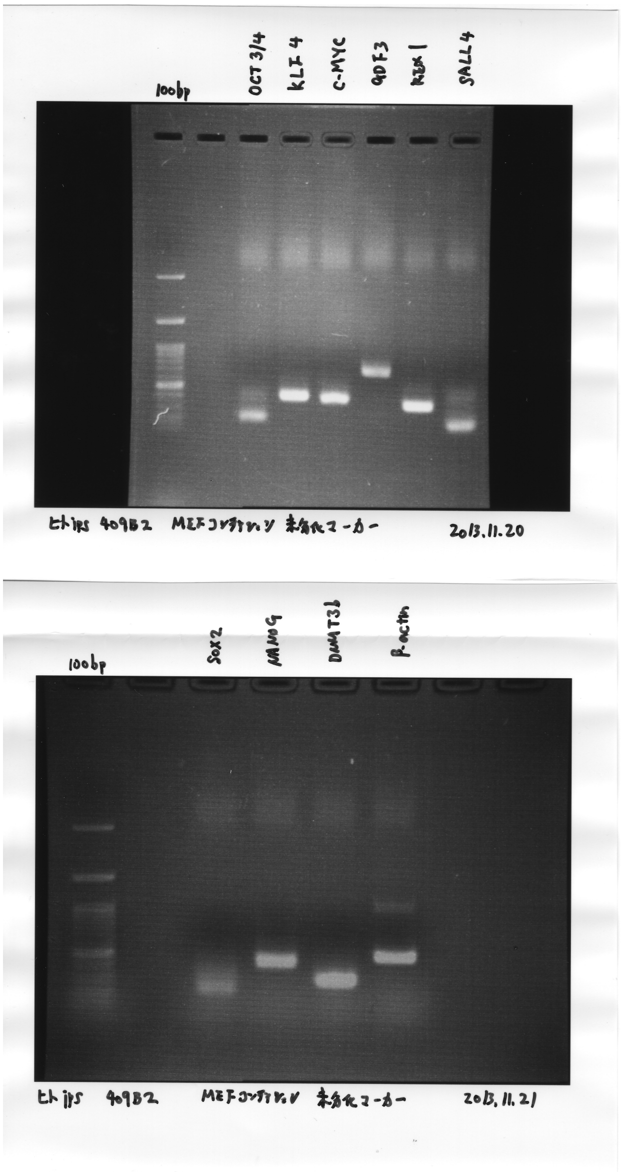

b

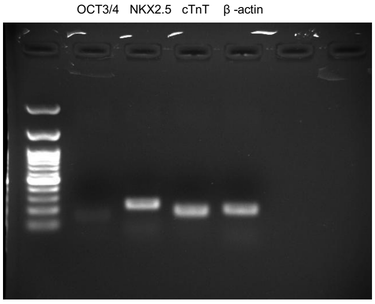

Figure S1. Full unedited gel. (a) Full-length images of the blots used for Figure 1d and (b) Figure S5c.

Figure S2

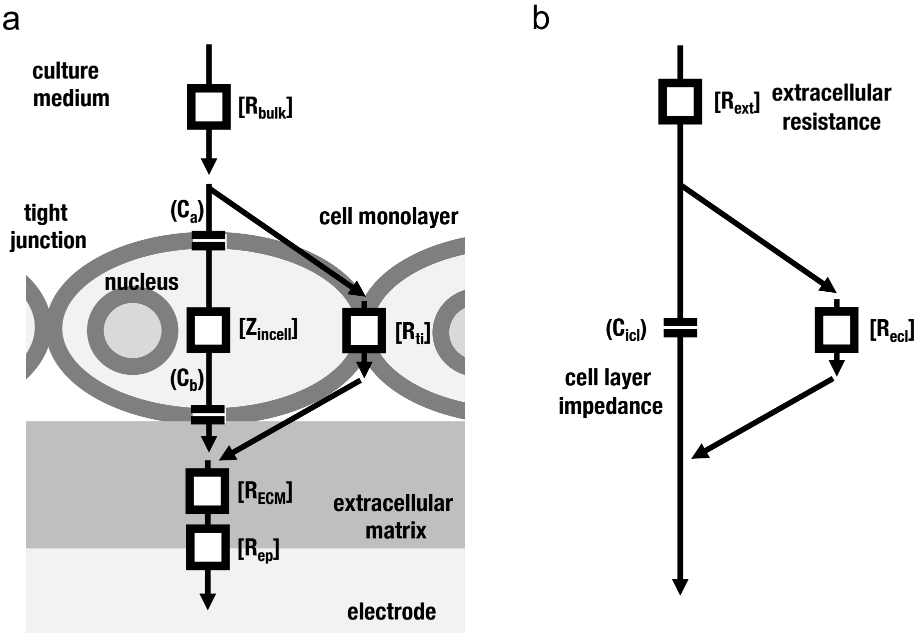

Electric resistance value in direct current (d.c) circuit [R_{DC}],
Voltage [V], intensity of electric current [I]
$V=R_{DC}I$ [Ohm's law]

Electric resistance value in alternating current (a.c) circuit [R_{AC}],
a.c voltage [ΔV],
a.c intensity of electric current [ΔI]
$\Delta V=R_{AC}\Delta I$
$\underline{R_{AC}}=\Delta V/\Delta I$= Impedance values [Z]

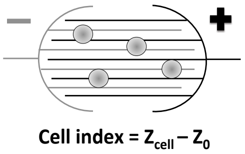

(a) : Factors affecting Z
Resistance of the medium [R_{bulk}], Resistance of the tight junction [R_{ti}], Capacitance of the apical cell membrane (C_a), Capacitance of the basal cell membrane (C_b), impedance of intracellular element (Z_{incell}), Resistance of extracellular matrix [R_{ECM}], Resistance of electrode plate (R_{ep})
(b) : Factor model affecting Z
Resistance of the extracellular element [R_{ext}], Resistance of the intercellular element [R_{ecl}], Capacitance of the cell monolayer (C_{icl})

Figure S2. A circuit diagram for calculating the impedance values of hiPSCs and differentiated cells derived from hiPSCs. (a) A configuration diagram of energizing circuit penetrating single cell layer of hiPSC under adhesion culture conditions. (b) A model diagram showing the method for calculating the impedance value of hiPSC under adhesive culture conditions.

Figure S3

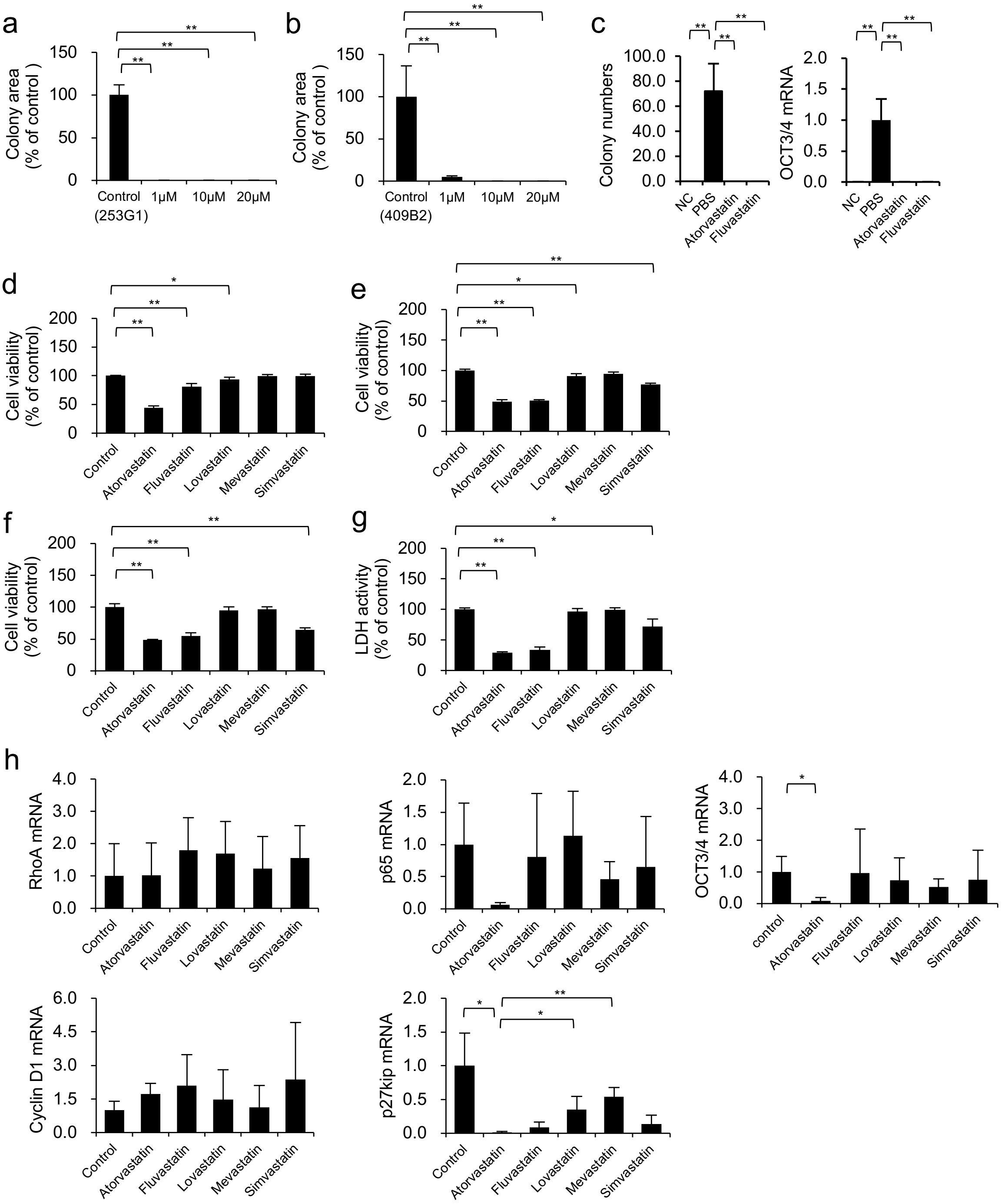

Figure S3. Cell assays and mRNA expression analysis. (a, b) Growth and viability assays of hiPSC lines 253G1 (a) and 409B2 (b) after 48 h of culture in the presence of 1, 10, 20 μM Atorvastatin. The cells were stained for AP. Colony areas per well were measured. The relative values are indicated. n = 3. Data represent mean ± S.D. *P < 0.05, **P < 0.01. (c) Left panel, the number of hiPSC colonies that were subjected to AP staining 4 days after the re-seeding of hiPSCs cultured under the same conditions as the teratoma experiments (Fig 2g) on MEF feeder—not mouse testis—and colony number/well were analyzed. n = 6. Data represent mean ± S.D. **P < 0.01. Right panel, a quantitative real-time PCR was performed to detect OCT3/4 mRNA (4 days after the re-seeding of hiPSCs cultured under the same conditions as the teratoma experiments [Fig 2g]) on MEF feeder—not mouse testis—and OCT3/4 mRNA expression level/well was analyzed. n = 6. Data represent the mean ± S.D. **P < 0.01. (d) Cell proliferation assays using Cell Counting Kit. (e) Cell count Reagent SF assay. (f) Assay of Neutral Red uptake. hiPSCs were analyzed after 48 h of culture in the presence of 20 μM Atorvastatin, Fluvastatin, Lovastatin, Mevastatin or Simvastatin. Each measurement was obtained using a microplate reader. (n = 3). Data represent mean ± S.D. *P < 0.05, **P < 0.01. (g) LDH assay of cytotoxicity in hiPSCs after 48 h of culture in the presence of 20 μM Atorvastatin, Fluvastatin, Lovastatin, Mevastatin or Simvastatin. Each measurement was obtained using a microplate reader. n = 3. Data represent mean ± S.D. *P < 0.05, **P < 0.01. (h) A quantitative real-time PCR analysis of RhoA, Cyclin D1, p65, p27kip and OCT3/4 mRNA in hiPSCs (201B7) after 24 h of culture in the presence of 10 μM Atorvastatin, Fluvastatin, Lovastatin, Mevastatin or Simvastatin. Data represent mean ± S.D. *P < 0.05.

Figure S4

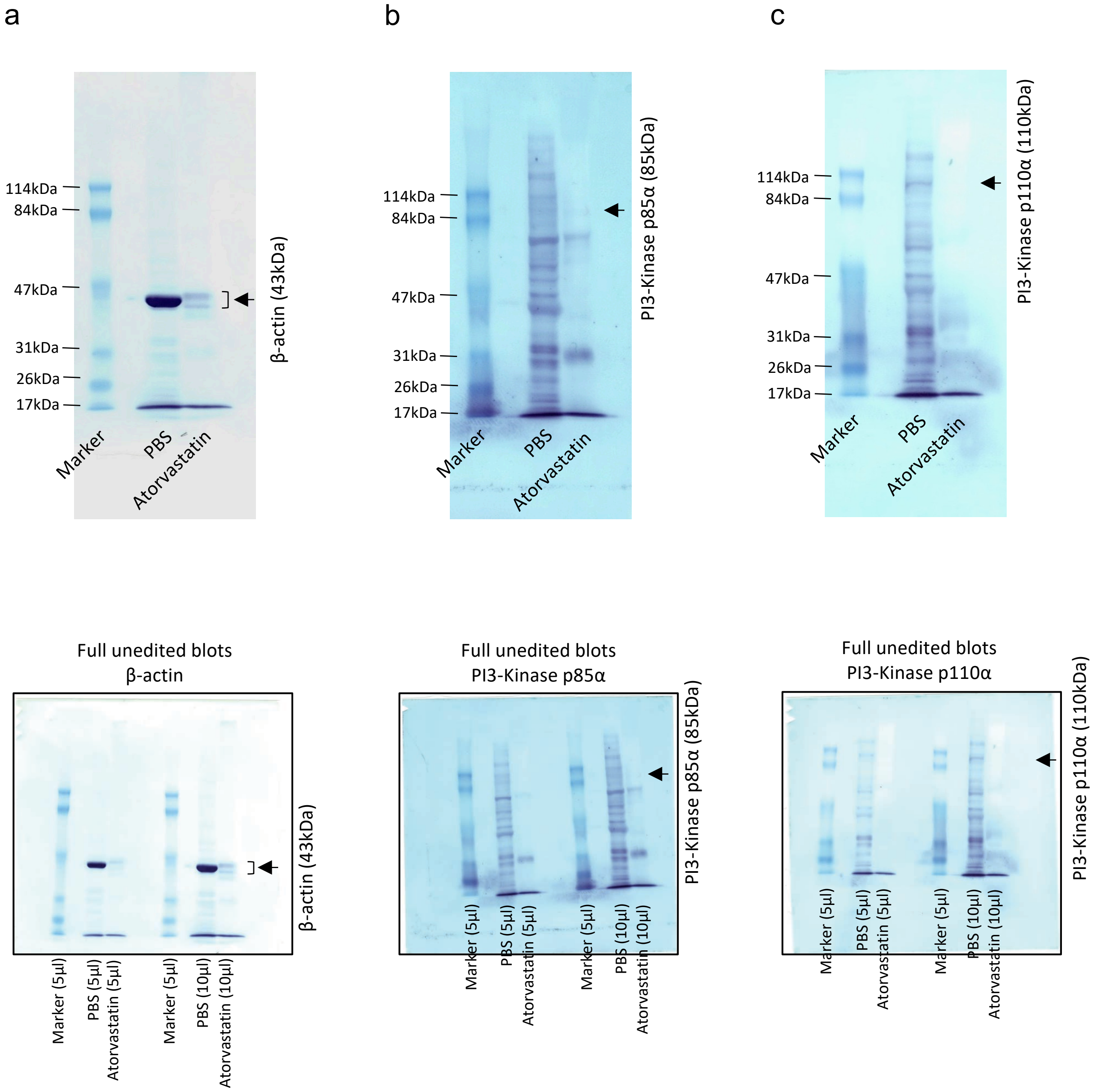

Figure S4. Western blot assays of hiPSCs (201B7) after 24 h of culture in the absence or presence of 20 µM Atorvastatin. (a) Top panel, full-length image of β-actin protein band photo sensitively detected on membrane by coloring. Bottom panel, Full unedited blots: Marker and β-actin. The same pair of samples flowed to the left (5 µl) and right (10 µl) of the membrane. (b) Top panel, full-length image of PI3-Kinase p85α protein band photo sensitively detected on membrane by coloring. PI3-Kinase p85α. Bottom panel, Full unedited blots: Marker and PI3-Kinase p85α. The same pair of samples flowed to the left (5 µl) and right (10 µl) of the membrane. (c) Top panel, full-length image of PI3-Kinase p110α protein band photo sensitively detected on membrane by coloring. PI3-Kinase p110α. Bottom panel, Full unedited blots: Marker and PI3-Kinase p110α. The same pair of samples flowed to the left (5 µl) and right (10 µl) of the membrane.

Figure S5

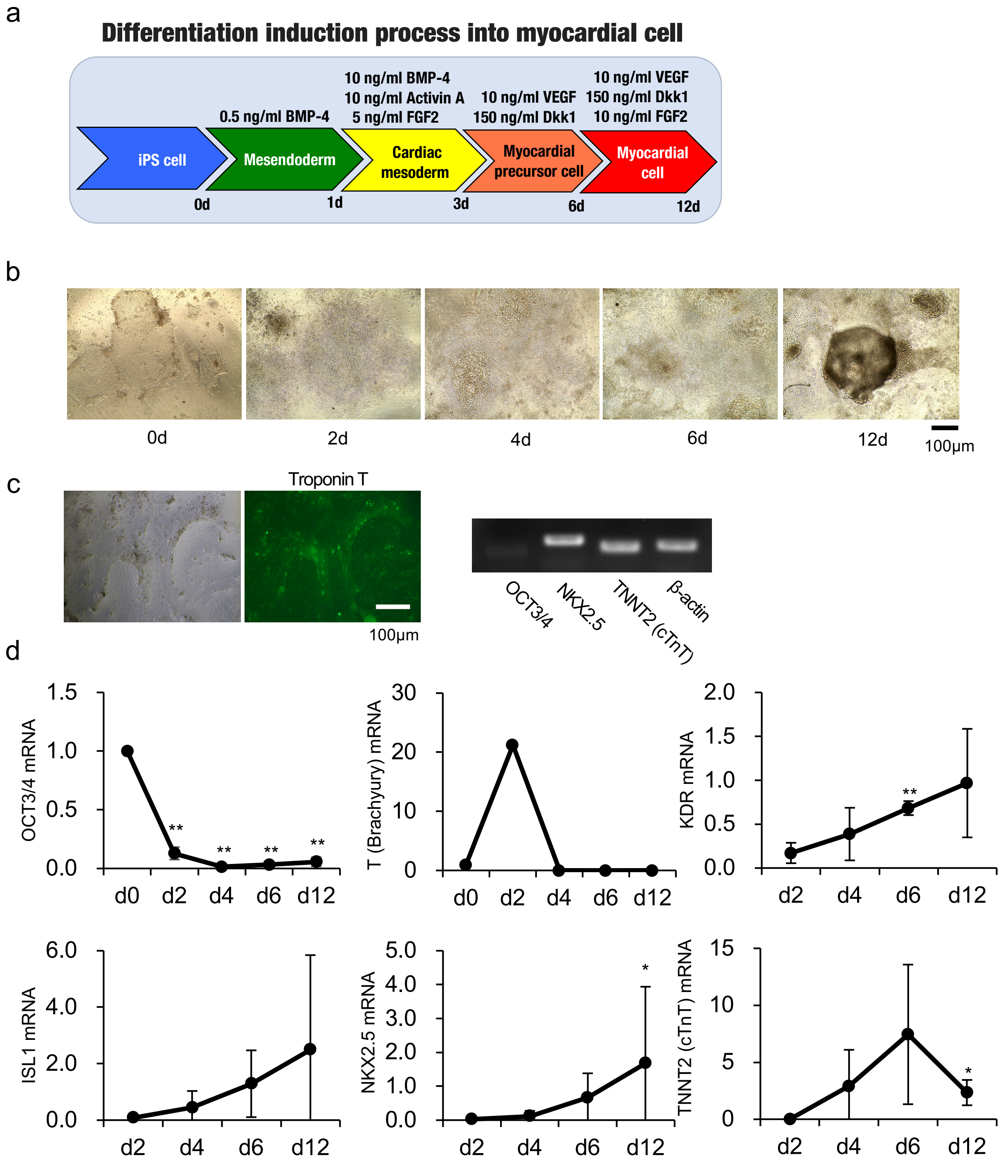

Figure S5. The method for inducing the differentiation of myocardial cells from hiPSCs (201B7), and a cellular characteristic of the differentiation stage. (a) A general myocardial cell differentiation induction protocol is shown. Representative medium addition factors and incubation period are shown. Differentiated cells at each stage are shown. (b) Microphotographs of 0, 2, 4, 6 and 12 d are shown after the initiation of differentiation induction. Scale bar = 100 μm. (c) Immunohistochemical staining method for Troponin T in myocardial cells differentiated from hiPSCs. Optical microscope images are shown (left is an optical microscope image, right is a fluorescence microscope image). Scale bar = 100 μm. Light panel, RT-PCR assay of OCT3/4, NKX2.5 and TNNT2 (cTnT) in myocardial cells differentiated from hiPSCs. (d) The mRNA expression levels of OCT3/4, T (Brachyury), KDR, ISL1, NKX2.5, TNNT2 (cTnT) at 0, 2, 4, 6 and 12 d after the initiation of differentiation induction are shown. The expression was calculated using the ΔΔCt method. The expression of the target gene was corrected by the expression of the housekeeping gene. The relative values are indicated. n = 3. Data represent the mean ± S.D. *$P < 0.05$, **$P < 0.01$.

Figure S6

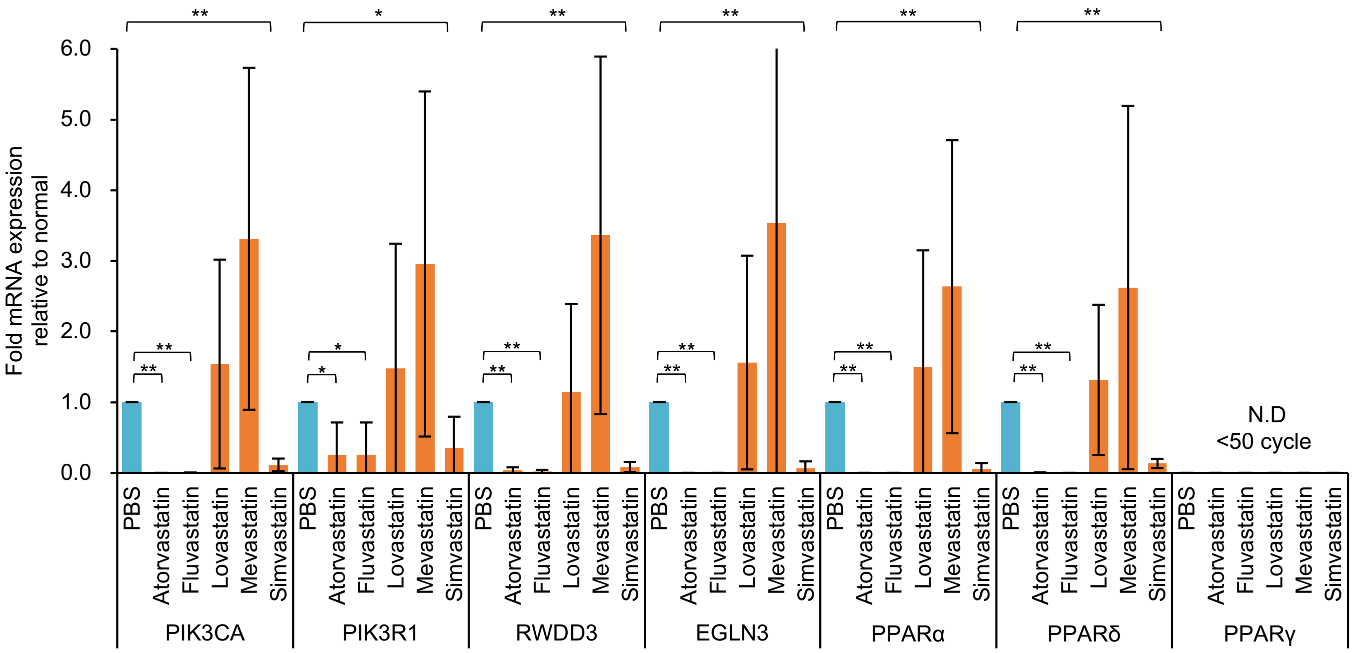

Figure S6. The PI3K/AKT, HIF1α, NF-κB and PPAR signaling are involved in the effect of Atorvastatin on hiPSCs. cDNA was synthesized using hiPSCs (201B7) administered PBS for 24 h and hiPSCs (201B7) administered 20 µM Atorvastatin, Fluvastatin, Lovastatin, Mevastatin and Simvastatin for 24 h. The mRNA expression level was calculated using the ΔΔCt method. The expression of the target gene was corrected by the expression of the housekeeping gene. First and second panels from the left, a quantitative real-time PCR analysis of the protein complex of PI3K. n = 4. Data represent mean ± S.D. $^{**}P < 0.01$. Third and fourth panels from the left, a quantitative real-time PCR analysis of HIF1α and NF-κB signaling. n = 4. Data represent the mean ± S.D. $^{**}P < 0.01$. 1st, 2nd and 3nd panels from the right, a quantitative real-time PCR analysis of PPARs signaling. n = 4. Data represent the mean ± S.D. $^{**}P < 0.01$.

Figure S7

a

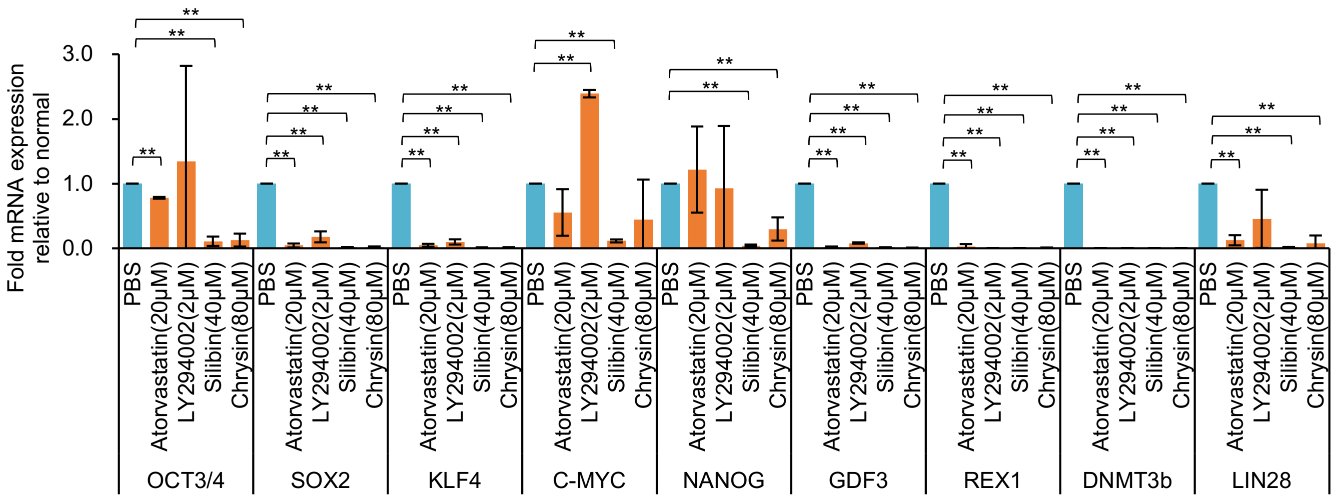

b

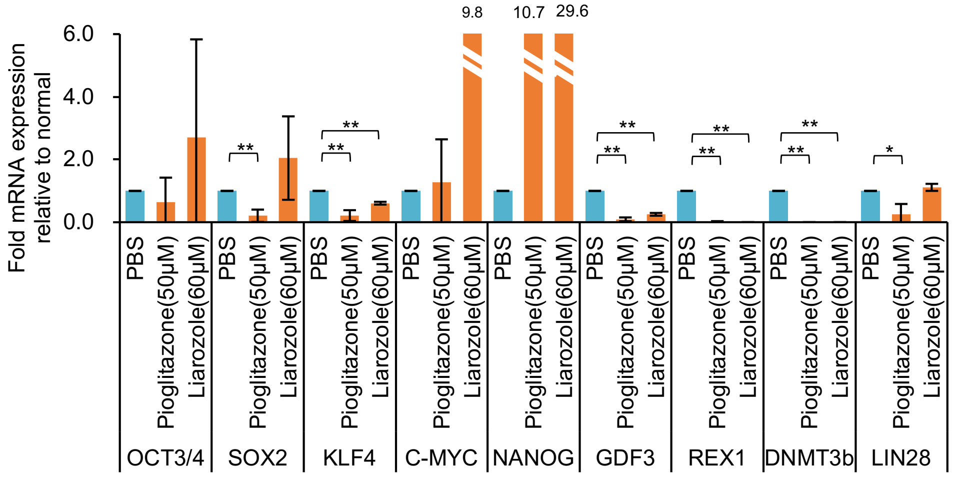

Figure S7. Effect of various drugs on the expression of undifferentiated marker mRNA of hiPSC. (a) The residual state of undifferentiated iPSCs are involved in the effect of Atorvastatin, LY294002, Silibin and Chrysin on hiPSCs. cDNA was synthesized using hiPSCs (201B7) administered PBS for 6 h and hiPSCs (201B7) administered 20 μM Atorvastatin, 2μM LY294002, 40 μM Silibin and 80 μM Chrysin for 6 h. The expression was calculated using the ΔΔCt method. The expression of the target gene was corrected by the expression of the housekeeping gene. A quantitative real-time PCR analysis of undifferentiated marker. n = 3. Data represent the mean ± S.D. **P < 0.01. (b) The residual state of undifferentiated iPSCs are involved in the effect of Pioglitazone and Liarozole on hiPSCs. cDNA was synthesized using hiPSCs (201B7) administered PBS for 6 h and hiPSCs (201B7) administered 50 μM Pioglitazone and 60 μM Liarozole for 6 h. The expression was calculated using the ΔΔCt method. The expression of the target gene was corrected by the expression of the housekeeping gene. A quantitative real-time PCR analysis of undifferentiated markers. n = 3. Data represent the mean ± S.D. *P < 0.05, **P < 0.01.

Figure S8

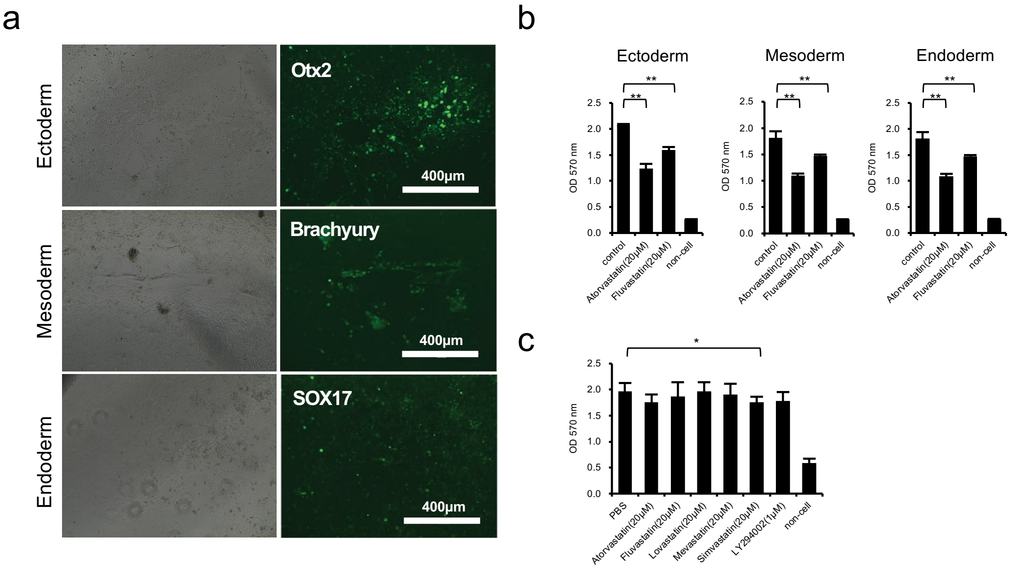

Figure S8. The effect of statins on the viable cell activity of endoderm, mesoderm, ectoderm and myocardial cells. (a) Top panel, Immunohistochemical staining of Otx2 on ectoderm differentiated from hiPSCs. Middle panel, Immunohistochemical staining of Brachyury in mesoderm differentiated from hiPSCs. Lower panel, Immunohistochemical staining of SOX17 in endoderm differentiated from hiPSCs. Optical microscope images are shown (left is an optical microscope image, right is a fluorescence microscope image). Scale bar = 400 µm. (b) Left panel, cell viability assays (MTT assay) of ectoderm differentiated from hiPSCs (201B7) after 48 h of culture in the presence of 20 µM Atorvastatin and 20 µM Fluvastatin. Each measurement was performed using a microplate reader. The relative values are indicated. n = 4. Data represent the mean ± S.D. **P < 0.01. Middle panel, cell viability assays (MTT assay) of mesoderm differentiated from hiPSCs (201B7) after 48 h of culture in the presence of 20 µM Atorvastatin and 20 µM Fluvastatin. Each measurement was performed using a microplate reader. The relative values are indicated. n = 4. Data represent the mean ± S.D. **P < 0.01. Right panel, cell viability assays (MTT assay) of endoderm differentiated from hiPSCs (201B7) after 48 h of culture in the presence of 20 µM Atorvastatin and 20 µM Fluvastatin. Each measurement was performed using a microplate reader. The relative values are indicated. n = 4. Data represent the mean ± S.D. **P < 0.01. (c) Cell viability assays (MTT assay) of myocardial cells differentiated from hiPSCs (201B7) after 48 h of culture in the presence of 20 µM Atorvastatin, Fluvastatin, Lovastatin, Mevastatin, Simvastatin and 1 µM LY294002. Each measurement was performed using a microplate reader. The relative values are indicated. n = 6. Data represent the mean ± S.D. *P < 0.05.
