# Supplementary material for: Atorvastatin Inhibits the HIF1α-PPAR Axis, Which Is Essential for Maintaining the Function of Human Induced Pluripotent Stem Cells
Source: Mol Ther. 2018 Jun 19;26(7):1715–34. doi: 10.1016/j.ymthe.2018.06.005 (PMC6036234; doi:10.1016/j.ymthe.2018.06.005)
Supplement: Document S2. Article plus Supplemental Information [file mmc2.pdf]

Molecular Therapy
Original Article

# Atorvastatin Inhibits the HIF1α-PPAR Axis, Which Is Essential for Maintaining the Function of Human Induced Pluripotent Stem Cells

Yoshiki Nakashima,[1,2,3] Chika Miyagi-Shiohira,[3] Hirofumi Noguchi,[3] and Takeshi Omasa[1,2]

[1]Faculty of Bioscience and Bioindustry, Tokushima University, Tokushima 770-8506, Japan; [2]Department of Material and Life Science, Graduate School of Engineering, Osaka University, Yamadaoka, Suita, Osaka 565-0871, Japan; [3]Department of Regenerative Medicine, Graduate School of Medicine, University of the Ryukyus, Nishihara-cho, Okinawa 903-0215, Japan

**We herein report a novel mechanism of action of statin preparations using a new drug discovery method. Milk fat globule-EGF factor 8 protein (MFG-E8) was identified from the secretory component of mouse embryonic fibroblast (MEF) as a cell adhesion-promoting factor effective for screening active cellular agents of human induced pluripotent stem cells (hiPSCs) *in vitro* using electrochemical impedance. Our analyses showed that atorvastatin did not cause death in myocardial cells differentiated from hiPSCs but reduced the pluripotent cell survival *in vitro* when using serum- and albumin-free media, and inhibited the ability to form teratomas in mice. This result could have been already the cytopathic effect of atorvastatin, and complete elimination of hiPSCs was confirmed in the xenotransplantation assay. The administration of atorvastatin to hiPSCs caused the expression of hypoxia inducible factor (HIF)1α mRNA to be unchanged at 6 hr and downregulated at 24 hr. In addition, the inhibition of the survival of hiPSCs was confirmed by HIF1α-peroxisome proliferator-activated receptor (PPAR) axis inhibition. These results suggest that the addition of atorvastatin to hiPSC cultures reduces the survival of pluripotent cells by suppressing the HIF1α-PPAR axis. In summary, the HIF1α-PPAR axis has an important role in maintaining the survival of pluripotent hiPSCs.**

## INTRODUCTION

The persistence of pluripotent cells in cell-based medicinal products derived from human induced pluripotent stem cells (hiPSCs)[1,2] is a potential risk for oncogenesis after transplantation into patients.[3,4] hPSCs have two unique properties: self-renewal, which is the ability to proliferate indefinitely while maintaining their cellular identity; and pluripotency, which is the ability to differentiate into all cell types that comprise the embryo proper. These traits make hPSCs promising for future application in regenerative medicine;[5] however, these same traits also make them potentially tumorigenic[6,7] and currently hinder the fulfillment of their clinical potential.[4] The specific problem associated with the medical use of cells differentiated from cultured hPSCs is the concern that some may not have completed differentiation. In this case, pluripotent hPSCs may be inadvertently transplanted along with differentiated cells. Therefore, pluripotent stem cells with a low risk for tumorigenesis are being developed.[8,9]

It was previously reported that hPSCs have a unique requirement for carbohydrate, amino acid, or lipid metabolism that is dependent on glucose, lactate, methionine, or oleic acid. Inhibitors of the ATP supply, such as S-adenosylmethionine, or inhibitors of oleate synthesis induce cytotoxicity and may selectively eliminate hPSCs.[10–12] Although these reports may be very valuable, hPSCs cultured in glucose-, methionine-, or oleic acid-free media do not differentiate. In addition, it is not feasible to recreate these restrictive environments *in vivo*, as would be necessary in regenerative medicine treatments. An alternative approach to reducing hPSC pluripotency that is receiving more attention is the use of statins. Statins[13] are orally administered competitive inhibitors of 3-hydroxy-3-methyl-glutaryl-coenzyme A (HMG-CoA) reductase, an enzyme that catalyzes the conversion of HMG-CoA to mevalonic acid;[14] their activity thus reduces the endogenous synthesis of cholesterol. They are effective and safe drugs that are widely prescribed in cholesterol-lowering therapy. Statins also have additional effects, such as the nitric oxide-mediated promotion of new blood vessel growth,[15] stimulation of bone formation,[16,17] and the reduction of both plasma low-density lipoprotein (LDL)[18] and C-reactive protein (CRP) levels to provide early clinical benefits in drug therapy.[19,20] The inhibition of cholesterol synthesis also leads to the reduced formation of intermediates, such as geranylgeranyl-diphosphate and farnesyl-diphosphate, which play a role in post-translational modification of proteins and in cell signaling.[21] In light of the widespread effects and consequences of statin treatments described above, the present study was performed to determine whether or not statins might be an effective means of removing residual undifferentiated hPSCs from cell cultures.

Received 3 October 2017; accepted 7 June 2018;
https://doi.org/10.1016/j.ymthe.2018.06.005.

**Correspondence:** Yoshiki Nakashima, DDS, PhD, Department of Regenerative Medicine, Graduate School of Medicine, University of the Ryukyus, 207 Uehara, Nishihara-cho, Okinawa 903-0215, Japan.
**E-mail:** nakasima@med.u-ryukyu.ac.jp


This is an open access article under the CC BY license (http://creativecommons.org/licenses/by/4.0/).

In this study, it was first necessary to find a method to scientifically evaluate the removal of undifferentiated hPSCs *in vitro*. Currently, the most reliable way to evaluate the removal of undifferentiated hPSCs is a mouse teratoma formation test (an animal experiment). However, experimental animals and a 10-week period are required for this experiment. Because undifferentiated hPSCs form colonies during culturing, the quality of hPSCs can be controlled by examining the shapes of colonies using an optical microscope.[22] However, this method is not suitable for investigating the removal of the undifferentiated hPSCs contained in differentiated cells. On the other hand, other research methods, such as analyses of cell viability, cell proliferation, and cell motility, have been developed using hPSCs or normal cells. These methods are frequently used for new drug development using high-throughput screening (HTS), studies on the influences of chemical substances, biosensors for measuring cell movement, the 3-(4,5-dimethylthiazol-2-yl)-2,5-diphenyltetrazolium bromide (MTT) method, the neutral red method, and ATP measurement and methods. Furthermore, PCRs have been used to analyze the expression of markers of undifferentiated hPSCs. However, these methods take time to perform, and the procedure is very complicated. Furthermore, continuous measurement cannot be performed. However, there is an impedance measurement method that uses microelectrodes that can easily detect cell behavior in real time.[23] It is possible to measure the behavior of the cell indirectly by measuring the changes in impedance caused by the adhesion, extension, and proliferation of cells on the electrode.

Unlike common cells, it is known that hPSCs do not adhere to uncoated culture dishes, and that this easily causes cell death.[24,25] The common impedance measurement method in which cells adhere to electrodes cannot be used to directly measure the impedance of hPSCs cultured using existing culture methods. The results of research on a new technique for culturing hPSCs under uncoated conditions are shown in Figure 1. The development of a method and a device to measure the electrochemical impedance of electrical signals over time from iPSCs (as well as cardiomyocytes and other cell types differentiated from iPSCs) is very important for drug discovery screening using iPSCs and for elucidating disease mechanisms. We started this study to measure electrochemical impedance with the aim of developing a method of acquiring electrical signals from hiPSCs for industrial application. Through this technique, it was discovered that some of the drugs mentioned in this study inhibited the survival of hiPSCs.

## RESULTS

### MFG-E8 Enhances the Sensitivity of the Electrochemical Impedance Measurement Method for hiPSCs and Cardiomyocytes Differentiated from hiPSCs

Generally, a culture dish with a special coating is necessary for culturing hiPSCs. Thus, it was difficult to obtain electrical information, because direct cell adhesion to the electrode plate is indispensable for such experiments. In order to investigate the survival activity of hiPSCs, we attempted to perform uncoated culturing, which would allow the hiPSCs to directly adhere to the electrode plate (Figure 1). To systematically assess the conditions in hiPSC cultures, we evaluated cell attachment using electrochemical impedance values, calculated as the interchange electrical resistivity in cells.[23,26] The principle of the electrochemical impedance measurement method is shown in Figure S2. Mouse embryonic fibroblast (MEF)-conditioned medium (MEF-CM) was prepared using Essential 8 medium, as described previously.[27] We examined the ability of medium conditioned by MEF-CM to promote cell attachment in culture dishes (Figures 1A and 1B) and impedance-measuring wells (Figure 1C). To evaluate the electrochemical impedance, we measured the alternating electric resistance value (RAC) between the medium and the electrode plate to which the cells adhered: Impedance values (Z). [Factors affecting Z] were then replaced by the formula of the [Factor model affecting Z] (Figures S2A and S2B).[23] This CM contains no components for inducing hiPSC differentiation (Figure 1D). Therefore, we used an impedance measuring device with MEF-CM to analyze the difference between two states of hiPSCs, e.g., differentiated and undifferentiated. As a result, the MEF-CM increased the sensitivity of impedance values (Figure 1E). This result indicates that MEF-CM contains components that promote the uncoated culturing of hiPSCs.

First, molecular weight fractionation of MEF-CM was performed using 10k, 30k, 50k, and 100k filters, or a change of the MEF-CM content to 1/1, 1/2, or 1/4 of the content of the assay medium. The results showed significant variability in the ability of the hiPSCs to adhere to non-coated plates. However, the cell adhesion-promoting effect was not found in MEF-CM prepared with fetal bovine serum (FBS)-free DMEM. When a protein component analysis was performed using the liquid chromatography tandem-mass spectrometry (LC/MS/MS) measurement method,[28] MEF-CM prepared in FBS-free DMEM contained uncharacterized aarF domain-containing protein kinase 2 (ADCK2). In our experiments, human ADCK2 (CUSABIO CSB-YP772029HU) did not show a cell adhesion-promoting effect on hiPSCs. Second, to identify the cell adhesion active component in MEF-CM, we carried out assays using recombinant proteins. Furthermore, the promotion of the cell adhesion of hiPSCs was not confirmed in medium containing dissolved extracellular matrix components, human laminin-111 (L4544; Sigma), human laminin-322 (ECHEOT004; ReproCELL), human secreted protein, acidic and rich in cysteine (SPARC) (941-SP-050; R&D), human Glypican 2 (2304-GP-050; R&D), human Glypican 4 (ATGEN ATGP2616), human Thioredoxin-1 (1970-TX-500; R&D), human cellular repressor of E1A-stimulated genes 1 (CREG1) (2380-CR-025; R&D), human collagen IV (354245; Corning), human nidogen (2570-ND; R&D), or mouse heparan sulfate proteoglycan (HSPG) (H4777; Sigma) (data not shown). We reduced the number of proteins that had the potential to promote the cellular adhesion of MEF-secreted hiPSCs by subtracting the protein components expressed by hiPSCs[29] from the protein components expressed by MEF.[30] Furthermore, commercially available recombinant proteins that had the possibility of promoting cell adhesion were selected using the ExPASy (Expert Protein Analysis System) proteomics server protein database (https://www.expasy.org/). Finally, we found that the milk fat globule-EGF factor 8 protein (MFG-E8) was one such component. Supplementation of

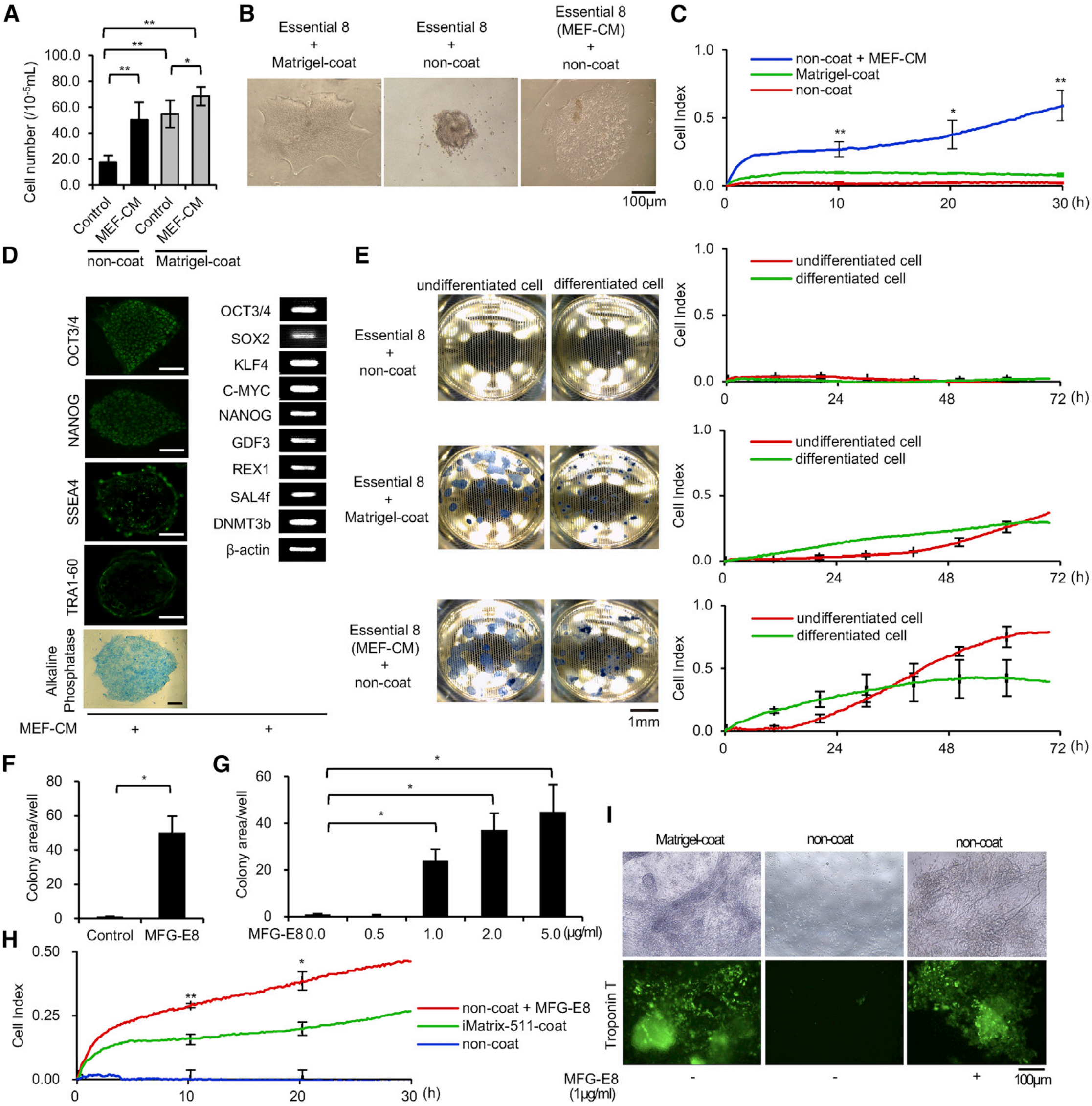


**Figure 1. Effectiveness of MEF-CM and MFG-E8 in Stimulating hiPSC Adhesion**

(A) Cell proliferation assay. The number of viable hiPSCs (409B2) was counted after 4 days in the absence or presence of MEF-CM and the absence or presence of a Matrigel coat. n = 3. Data represent mean ± SD. *p < 0.05; **p < 0.01. (B) hiPSCs (409B2) after 4 days of culture in the absence or presence of MEF-CM and the absence or presence of a Matrigel coat; optical microscope images are shown. Scale bar, 100 μm. (C) Electrochemical impedance measurements of hiPSCs (409B2) after 30 hr of culture in the absence or presence of MEF-CM and the absence or presence of a Matrigel coat. The online data of measurements are displayed as lines, and the data sampled offline are displayed as the measured. n = 3. Data represent mean ± SD. *p < 0.05; **p < 0.01. (D) hiPSCs (409B2) cultured using MEF-CM on a Matrigel coat for 10 passages. Left panels: immunofluorescence and AP staining for pluripotency markers are shown. Scale bars, 100 μm. Right panel: an RT-PCR analysis of human embryonic stem cell markers is shown. (E) Electrochemical impedance measurements of undifferentiated (normal) hiPSCs (409B2) and differentiated hiPSCs (409B2) after 72 hr of culture in the absence or presence of MEF-CM and the absence or presence of a Matrigel coat. Left panels: the base of the electrode plate is shown in the image. Right panels: impedance values (cell index) are shown. The online data of measurements are displayed as lines, and the data sampled offline are displayed as the measured value. n = 3. Data represent mean ± SD. *p < 0.05. (F) Cell growth and viability assays of hiPSCs (201B7) after 4 days of culture in the absence or presence of 2 μg/mL MFG-E8; the cells are stained for AP. Colony areas per well were measured. The relative values are indicated. n = 3. Data represent mean ± SD. *p < 0.05. (G) Cell growth and viability assays of hiPSCs (201B7) after 4 days of culture in the absence or presence of 0.0, 0.5, 1.0, 2.0, and 5.0 μg/mL MFG-E8; the cells are stained for AP. Colony areas per well were measured. The relative values are indicated. n = 3. Data represent mean ± SD. *p < 0.05. (H) Electrochemical impedance measurements of hiPSCs (201B7) after 30 hr of culture in the absence or presence of 2.5 μg/mL MFG-E8 and the absence or presence of an iMatrix-511 coat. The online data of measurements are displayed as lines, and the data sampled offline are displayed as the measured value. n = 3. Data represent mean ± SD. *p < 0.05; **p < 0.01. (I) Assay of differentiation of myocardial cells from hiPSCs (201B7) after 10 days of culture in the absence or presence of 1.0 μg/mL MFG-E8 and the absence or presence of a Matrigel coat. n = 3. Top panels: optical microscope images are shown. Bottom panels: immunostaining for troponin T proteins is shown. Scale bar, 100 μm.

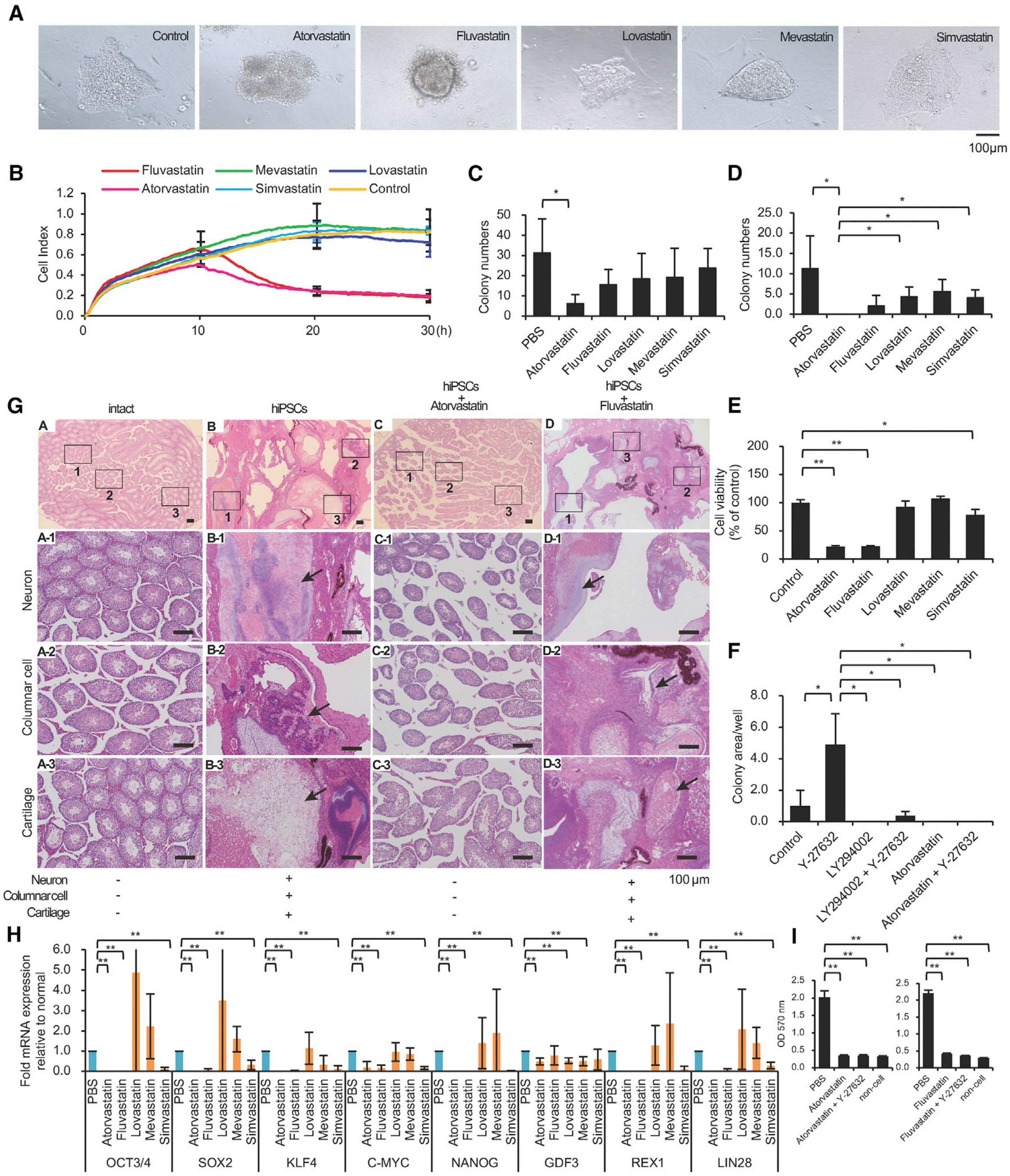

**Figure 2. Atorvastatin Reduces Cell Viability in hiPSC Cultures**

(A) hiPSCs (201B7) after 48 hr of culture in the presence of 20 μM atorvastatin, fluvastatin, lovastatin, mevastatin, or simvastatin. Optical microscope images are shown. Scale bar, 100 μm. (B) Electrochemical impedance measurements of hiPSCs (201B7) after 0–30 hr of culture in the presence of 10 μM atorvastatin, fluvastatin, lovastatin, mevastatin, or simvastatin. The online data of measurements are displayed as lines. n = 2. (C) Cell growth and viability assays of hiPSCs (201B7) after 48 hr of culture in the presence of 1 μM atorvastatin, fluvastatin, lovastatin, mevastatin, or simvastatin. The cells are stained for AP. Colony numbers per well were measured. n = 4. Data represent mean ± SD. $^*p < 0.05$. (D) Cell growth and viability assays of hiPSCs (201B7) after 48 hr of culture in the presence of 20 μM atorvastatin, fluvastatin, lovastatin, mevastatin, or simvastatin. The cells are stained for AP. Colony numbers per well were measured. n = 4. Data represent mean ± SD. $^*p < 0.05$. (E) MTT cell count assays. hiPSCs were analyzed after 48 hr of culture in the presence of 20 μM atorvastatin, fluvastatin, lovastatin, mevastatin, or simvastatin. Each measurement was obtained using a microplate reader (n = 3). Data represent mean ± SD. $^*p < 0.05$. (F) Cell growth and viability assays of hiPSCs (201B7) after 24 hr of culture in the presence of 30 μM Y-27632, 1 μM LY-294002, or 10 μM atorvastatin. The cells were stained for AP. Colony areas per well were measured. The relative values are indicated. n = 3. Data represent mean ± SD. $^*p < 0.05$. (G) Atorvastatin prevents teratoma formation. H&E staining of teratomas is shown. After 10 weeks, the teratomas contained a mixture of well-differentiated tissues,

(legend continued on next page)

the medium with >1 μg/mL MFG-E8 improved the cell growth and viability of hiPSCs in uncoated dishes (Figures 1F and 1G) and significantly increased electrical impedance measurements in the cultures (Figure 1H). Furthermore, myocardial cell differentiation was induced in cultures with medium supplemented with MFG-E8 in uncoated wells (Figure 1I). This result shows that the electrochemical impedance of hiPSC and hiPSC-induced myocardial cells can be measured using MFG-E8-supplemented medium.

### Atorvastatin Reduces the Survival of hiPSCs and Prevents Teratoma Formation

The electrochemical impedance measurement method can be used to rapidly screen the effects of drugs on hiPSCs and on cells that differentiate from hiPSCs. We found that atorvastatin (20 μM × 48 hr) and fluvastatin (20 μM × 48 hr) reduced colony formation in hiPSC cultures (Figure 2A). The effect on cell viability was evaluated as the electrochemical impedance. Among the statins tested, only atorvastatin (10 μM × 30 hr) and fluvastatin (10 μM × 30 hr) showed a potent inhibitory effect on the hiPSC survival (Figure 2B). Indeed, the number of alkaline phosphatase (AP) staining-positive colonies significantly decreased in the atorvastatin (1 μM × 48 hr)-treated cultures (Figure 2C), and AP-staining-positive colonies were not observed in atorvastatin (20 μM × 48 hr)-treated cultures (Figure 2D). In order to measure the amount of living cells, we used an MTT Cell Count Kit (Figure 2E) and Cell Count Reagent SF (Figure S3E) (formazan dye stains with dehydrogenase in the cell), a Cell Counting Kit (Figure S3D) (NADH in the cell stains WST-1), and an assay of the neutral red uptake (Figure S3F) (pigment incorporated in living cells). We used the Cytotoxicity LDH Assay kit (Figure S3G) (measuring the lactate dehydrogenase [LDH] activity released into the medium from the cells) to determine the amount of dead cells. We then investigated the effect of atorvastatin on iPSCs using LY294002,[31] an inhibitor of phosphoinositide 3-kinases (PI3Ks), and Y-27632, a Rho kinase/ROCK signaling inhibitor.[24,32] LY-294002 at 1 μM reduced the viability of hiPSCs after 24 hr, but the survival activity of hiPSCs was partially improved by the addition of Y-27632 at a concentration of 30 μM. Atorvastatin at 10 μM reduced the survival activity of hiPSCs after 24 hr, but the addition of Y-27632 at 30 μM did not improve the survival activity of hiPSCs (Figure 2F). One possible reason for this is that atorvastatin may affect the PI3K signaling pathways. Atorvastatin treatment resulted in a loss of hiPSCs not only in the 201B7 and 253G1 cell lines (Figure S3A), but also in the 409B2 cell line (Figure S3B).

Teratomas did not occur in the testes of mice not injected with hiPSCs (zero of five mice) and did occur in the testes of all mice injected with hiPSCs (four of four mice). Teratomas did not occur in the testes of mice injected with hiPSCs cultured with 20 μM atorvastatin for 48 hr (zero of three mice) and did occur in the testes of all mice injected with hiPSCs cultured with 20 μM fluvastatin for 48 hr (four of four mice) (Figure 2G). This result shows that atorvastatin can efficiently remove tumorigenic undifferentiated cells from hiPSC cultures. These findings indicated that atorvastatin-treated stem cells lost the capacity to initiate teratoma formation. We also conducted an *in vitro* experiment to measure the amount of remaining undifferentiated iPSCs. First, iPSCs were cultured in a test well (without iPSCs), PBS, atorvastatin (20 μM × 48 hr), or fluvastatin (20 μM × 48 hr). Next, in the test groups, hiPSCs were seeded again on the MEF feeder, and the colonies that were AP-positive on day 4 were measured (n = 6). hiPSCs treated with atorvastatin or fluvastatin did not colonize on MEF feeders (Figure S3C, left panel). In addition, by measuring the Oct3/4 mRNA expression level in a real-time PCR, we confirmed that hiPSCs treated with atorvastatin or fluvastatin did not survive on the MEF feeder (Figure S3C, right panel).

Atorvastatin is the most potent statin used in this study. Thus, it is not surprising that it showed an effect. However, fluvastatin has also been shown to clearly reduce the electrochemical impedance values in hiPSCs (Figure 2B). Simvastatin preferentially inhibited the mRNA expression level of the hiPSC undifferentiated marker gene; however, its effect was insufficient for lowering the mRNA expression level to below the limit of PCR-based detection (Figure 2H). Fluvastatin had the same effect as atorvastatin with regard to inhibiting the survival of hiPSCs in the presence of Y-27632 (Figure 2I). These results show that both atorvastatin and fluvastatin are highly potent agents for eliminating hiPSCs, which occurs by a homologous pharmacological mechanism.

### Measuring the Influence of Statins on Myocardial Cells Differentiated from hiPSCs

Assays were performed using myocardial cells induced from hiPSCs (Figures 5A–5D). Cell death assays showed that fluvastatin increased the rate of cell death in myocardial precursor cells induced from hiPSCs, while atorvastatin had no injurious effects compared with the control agent (Figure 3A). Cell-death-inducing effects in rat myocardial cells have been reported for fluvastatin,[33] but not atorvastatin.[34]

We next examined myocardial cells at different stages of induced differentiation (Figure S5) from hiPSCs by measuring electrochemical impedance in mesendoderm (Figure 3B), cardiac mesoderm

including cartilage, columnar cells, and neurons in tissues from mice injected with hiPSCs (201B7) or hiPSCs + 20 μM fluvastatin at 48 hr of culture (black arrows). Teratoma formation did not occur in mice after transplantation of hiPSCs + 20 μM atorvastatin after 48 hr of culture as with vehicle control-treated mice. Scale bars, 100 μm. (H) The residual state of undifferentiated iPSCs is involved in the effects of atorvastatin, fluvastatin, lovastatin, mevastatin, and simvastatin on hiPSCs. cDNA was synthesized using hiPSCs that had been treated with PBS for 24 hr and hiPSCs that had been treated with 20 μM atorvastatin, fluvastatin, lovastatin, mevastatin, and simvastatin for 24 hr. The expression was calculated using the ΔΔCt method. The expression of the target gene was corrected by the expression of the housekeeping gene. A real-time qPCR was performed to detect the undifferentiated marker. n = 4. The data represent the mean ± SD. **p < 0.01. (I) Cell viability assays (MTT assay) of hiPSCs (201B7) after 48 hr of culture in the presence of 20 μM atorvastatin and 25 μM Y-27632 (left panel), and 20 μM fluvastatin and 25 μM Y-27632 (right panel). Each measurement was obtained using a microplate reader. The relative values are indicated. n = 5. The data represent the mean ± SD. **p < 0.01.

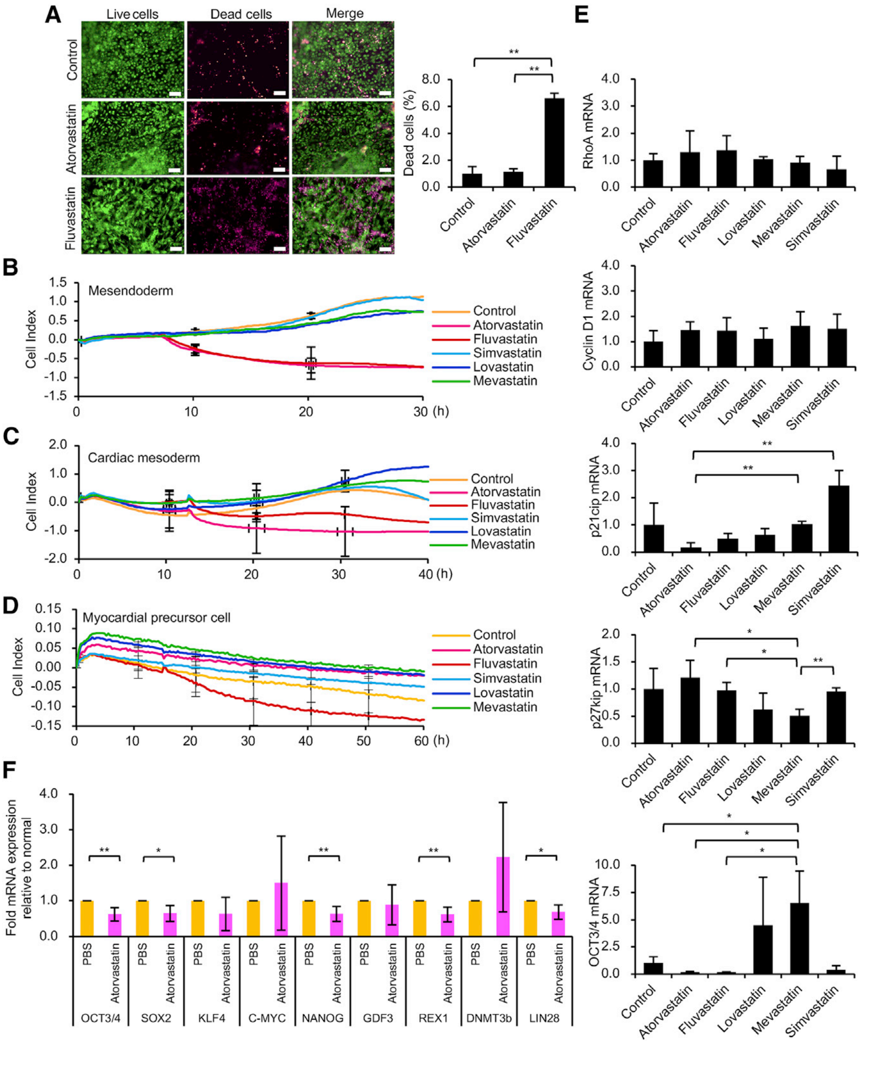


**Figure 3. Effect of Statins on hiPSC-Derived Myocardial Cells**

(A) Cell death assay of myocardial precursor cells differentiated from hiPSCs after 24 hr of culture in the presence of 10 μM atorvastatin and fluvastatin. Left panels: live cells are stained green, and dead cells are stained red. Right panel: the ratio of the areas of dead cells to live cells was measured. n = 3. Data represent mean ± SD. $^*p < 0.05$; $^{**}p < 0.01$. (B) Electrochemical impedance measurements of mesendoderm differentiated from hiPSCs after 0–30 hr of culture in the presence of 10 μM atorvastatin, fluvastatin, lovastatin, mevastatin, or simvastatin. The online data of measurements are displayed as lines. n = 2. (C) Electrochemical impedance measurements of cardiac mesoderm differentiated from hiPSCs after 0–40 hr of culture in the presence of 10 μM atorvastatin, fluvastatin, lovastatin, mevastatin, or simvastatin. The online data of measurements are displayed as lines. n = 2. (D) Fluvastatin treatment reduces the impedance values of myocardial precursor cells. Electrochemical impedance measurements of myocardial precursor cells differentiated from hiPSCs after 0–60 hr of culture in the presence of 10 μM atorvastatin, fluvastatin, lovastatin, mevastatin, or simvastatin. The online data of measurements are displayed as lines. n = 2. (E) A real-time qPCR analysis of RhoA, Cyclin D1, p21cip, p27kip, and OCT3/4 mRNA in myocardial precursor cells and myocardial cells differentiated from hiPSCs (201B7) after 24 hr of culture in the presence of 10 μM atorvastatin, fluvastatin, lovastatin, mevastatin, or simvastatin. Data represent mean ± SD. $^*p < 0.05$; $^{**}p < 0.01$. (F) The residual state of undifferentiated iPSCs are involved in the effect of atorvastatin on hiPSC-derived myocardial cells. cDNA was synthesized using hiPSC-derived myocardial cells that had been administered PBS for 6 hr and hiPSC-derived myocardial cells that had been administered 20 μM atorvastatin for 6 hr. The expression was calculated using the ΔΔCt method. The expression of the target gene was corrected by the expression of the housekeeping gene. A real-time qPCR analysis of the undifferentiated marker is shown. n = 3. Data represent the mean ± SD. $^*p < 0.05$; $^{**}p < 0.01$.

(Figure 3C), and myocardial precursor cells (Figure 3D). Our results indicated that fluvastatin, but not other statins, reduced the impedance values in the myocardial precursor cells (Figures 3A and 3D). Statins (20 μM) were allowed to act on myocardial cells differentiated from hiPSCs for 48 hr, and the viable cell activity was measured by an MTT assay. The results showed that neither atorvastatin nor fluvastatin inhibited the viable cell activity of myocardial cells (Figure S8C). Figures 3A and 3D show the results of experiments using myocardial precursor cells and demonstrate that fluvastatin's inhibition of viable cell activity remains. Experiments using myocardial cells with a higher stage of differentiation maturation showed that fluvastatin did not suppress viable cell activity (Figure S8C). Atorvastatin (20 μM) and fluvastatin (20 μM) were used for 48 hr in experiments using ectoderm, mesoderm, and endoderm differentiated from hiPSCs. The results of the MTT assay showed that atorvastatin and fluvastatin significantly inhibited the live-cell activity of ectoderm, mesoderm, and endoderm (Figure S8B). This result is consistent with the data obtained using electrochemical measurement methods (Figure 3B). This result may indicate that the susceptibility of cellular survival to atorvastatin differs during myocardial cell differentiation. However, we demonstrated that undifferentiated iPSCs remained in not only mesendoderm, cardiac mesoderm, and myocardial precursor cells, but also in myocardial cells, and the expression of OCT3/4 mRNA was observed (Figure S5D). The reduction in the impedance values of mesendoderm, cardiac mesoderm, and myocardial precursor cells might have been caused by residual undifferentiated iPSCs. Each statin (10 μM for 24 hr) was administered to myocardial precursor cells and myocardial cells differentiated from hiPSCs. To investigate the effect of statins on cell death of hiPSC-derived myocardial cells, we investigated mRNA expression focusing on the cell cycle of myocardial precursor cells and myocardial cell death. Myocardial cells remain in the G0 phase, and the cell cycle has stopped.[35] However, cell death occurs when the cell cycle is activated, such as by expression of Cyclin D1 and reduction of p21cip to suppress Cyclin

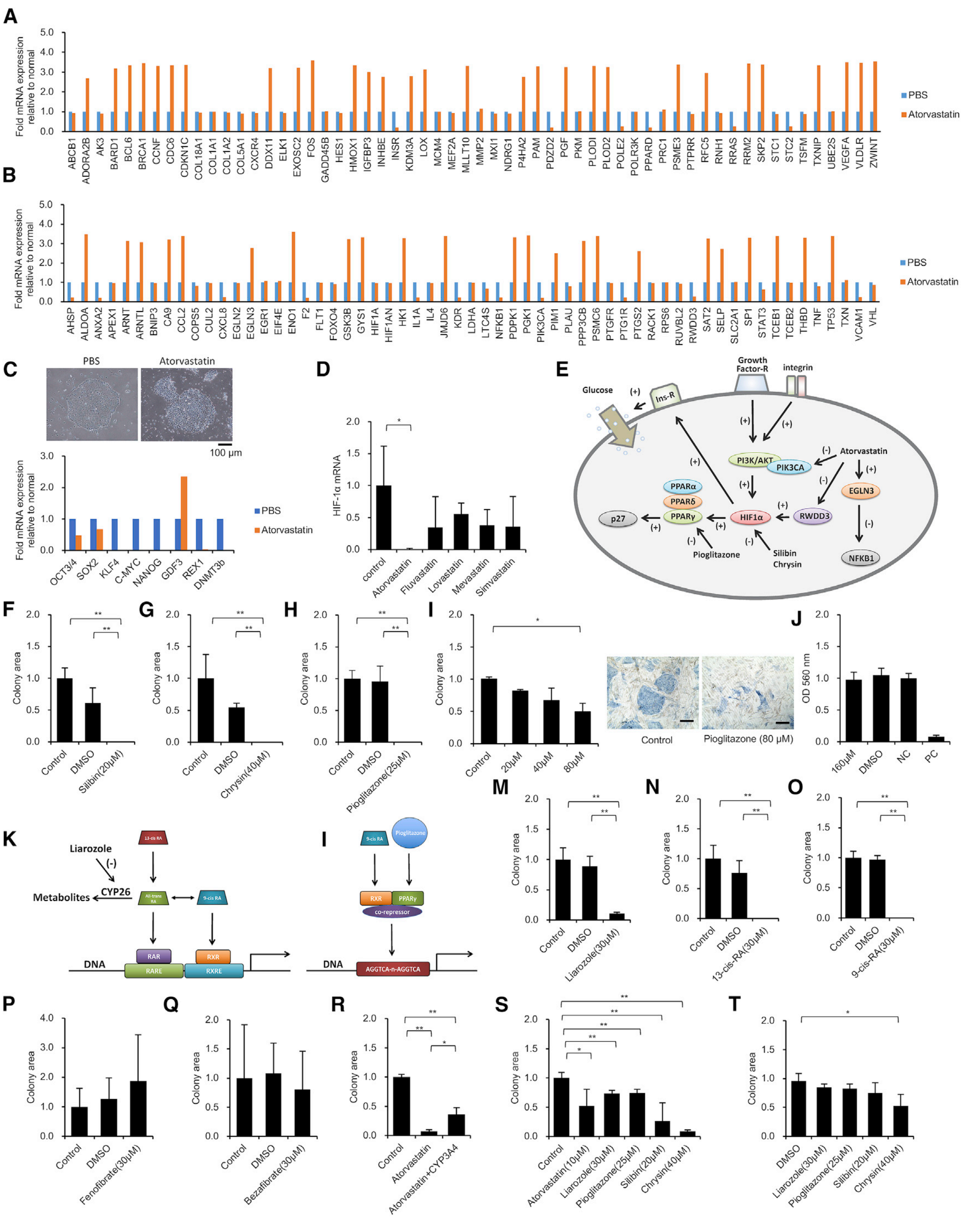


**Figure 4. The HIF1α-PPAR Axis Is Involved in the Effect of Atorvastatin on hiPSCs**

(A–C) cDNA was synthesized using hiPSCs (201B7) administered PBS for 6 hr and hiPSCs (201B7) administered 20 μM atorvastatin for 6 hr. The expression was calculated using the ΔΔCt method. The expression of the target gene was corrected by the expression of the housekeeping gene. (A) A real-time qPCR analysis of the hypoxia response (a qPCR array; GQH-HPX). n = 1. (B) A real-time qPCR analysis of HIF1 signaling (a qPCR array; GQH-HFT). n = 1. (C) hiPSCs (201B7) after 6 hr of culture in the absence or presence of 20 μM atorvastatin. Optical microscope images are shown (top). Scale bar, 100 μm. A real-time qPCR analysis of undifferentiated marker is shown. n = 1. (D) A real-time qPCR analysis of HIF1α mRNA in hiPSCs (201B7) after 24 hr of culture in the presence of 10 μM atorvastatin, fluvastatin, lovastatin, mevastatin, or simvastatin. Data represent mean ± SD. *$p < 0.05$. (E) A schematic diagram of the mechanism by which atorvastatin suppresses the survival activity of hiPSCs. The hiPSCs strengthen the

*(legend continued on next page)*

D1. However, p21cip is reported not to function in hiPSCs.[36] The Cyclin D1 mRNA expression level in myocardial precursor cells was significantly increased by the effect of atorvastatin and fluvastatin (10 μM for 24 hr) (data not shown). However, at the myocardial cell stage of differentiation, atorvastatin and fluvastatin did not increase the mRNA expression level of Cyclin D1 (Figure 3E). Atorvastatin and fluvastatin (10 μM for 24 hr) increased the RhoA mRNA expression level in hiPSC-derived myocardial precursor cells (Figure 3E). In an mRNA expression analysis of undifferentiated hiPSCs, atorvastatin (10 μM for 24 hr) significantly inhibited the mRNA expression level of p27 kip and OCT3/4 (Figure S3H). However, atorvastatin (10 μM for 24 hr) did not reduce the expression of p27 kip mRNA in myocardial precursor cells (Figure 3E). The p21 cip mRNA expression level was examined in myocardial precursor cells to investigate the mechanism through which fluvastatin induced cell death. The mRNA expression level of p21 cip of myocardial precursor cells was not only reduced by fluvastatin (10 μM for 24 hr), but also by atorvastatin (10 μM for 24 hr) (Figure 3E). Thus, it cannot be concluded that the forcible activation of the cell cycle by the increase of Cyclin D1 and the decreased expression of p21 cip on myocardial precursor cells is the major mechanism through which fluvastatin promotes cell death (Figures 3A and 3D). Atorvastatin and fluvastatin do not cause cell death in myocardial cells at an advanced stage of maturation (Figure S8C). These results indicate that the effects of atorvastatin and fluvastatin are not involved in the cell death of hiPSC-derived myocardial cells. We added 20 μM atorvastatin to the culture wells of the hiPSC-derived myocardial cells for 6 hr. We then examined changes in the mRNA expression levels of OCT3/4, SOX2, KLF4, C-MYC, NANOG, GDF3, REX1, DNMT3b, and LIN28 by a qPCR (Figure 3F). The results showed that atorvastatin significantly reduced the OCT3/4, SOX2, NANOG, REX1, and LIN28 mRNA expression levels of hiPSC-derived myocardial cells. This result indirectly indicates that atorvastatin reduces the possibility of teratoma formation remaining in hiPSC-derived myocardial cells.

### Atorvastatin Suppresses HIF Signaling

To investigate the cause of the inhibition of p27 kip and OCT3/4 gene expression by atorvastatin, we performed a gene expression analysis on genes associated with hypoxia-inducible factor 1 (HIF1). The effects of atorvastatin on the hypoxia response (a qPCR array; GQH-HPX) (Figure 4A) and HIF1 signaling response (a qPCR array; GQH-HFT) (Figure 4B) in undifferentiated hiPSCs were evaluated. PI3K is an enzyme consisting of a 110-kDa catalytic subunit and an 85-kDa control subunit. The phosphatidylinositol-4,5-bisphosphate 3-kinase catalytic subunit alpha (PIK3CA), also called the p110α protein, is a class of phosphatidylinositol 3-kinase (PI3K) catalytic subunit.[37,38] In recent years, PIK3CA has been reported to be a target mutant gene in cancer cells, as well as a gene controlling glutamine cell metabolism of cancer cells.[39–41] Atorvastatin strongly inhibited PIK3CA in undifferentiated hiPSCs (Figure S6), showing that atorvastatin suppresses the PI3K/AKT signaling[42] essential for the survival of hiPSCs. When the expression level of PI3-Kinase p85α and p110α protein were examined by western blotting, the amount of PI3-Kinase p110α protein decreased in hiPSCs supplemented with 20 μM × 24 hr of atorvastatin in the medium (Figures S4A–S4C). The expression of PI3-Kinase p85α protein was examined. However, the PI3-Kinase p85α protein could not be clearly detected in PBS-treated or atorvastatin-treated samples by western blotting. PI3K is a heterodimer

PI3K/AKT signal, which plays an important role in maintaining the survival through integrin and growth factor receptor. The intensity of the PI3K/AKT signal of hiPSCs reportedly varies depending on the culture medium and scaffold material.[112] Atorvastatin inhibits PIK3CA and RWDD3 of hiPSCs, and enhances EGLN3. Therefore, the PI3K/AKT and NF-κB signals are suppressed. Suppression of the PI3K/AKT signal reduces the expression of HIF1α. HIF1α promotes insulin receptor expression. Therefore, the ability to respond to insulin receptors, which is extremely important for maintaining the survival of hiPSCs, is decreased by suppressing HIF1α. The HIF1α-PPAR axis promotes the expression of p27kip. Atorvastatin inhibits the expression of PIK3CA and RWDD3, and promotes the expression of EGLN3 in a short time after administration. It therefore suppresses the PI3K/AKT signal and the survival activity of hiPSCs by inhibiting the HIF1α-PPAR axis. (F–H) Cell growth and viability assays of hiPSCs (201B7) after 48 hr of culture in the presence of 20 μM silibin (F), 40 μM chrysin (G), or 25 μM pioglitazone (H). The cells were stained for AP. Colony areas per well were measured. The relative values are indicated. n = 4. Data represent mean ± SD. **p < 0.01. (I) Cell growth and viability assays of hiPSCs (201B7) under co-culture conditions with human corneal epithelial cells after 48 hr of culture in the absence or presence of 20, 40, and 80 μM pioglitazone. The cells are stained for AP. Colony areas per well were measured. The relative values are indicated. n = 3. Data represent mean ± SD. *p < 0.05. Optical microscope images are shown (right panels). Scale bars, 100 μm. (J) Cytotoxicity assays of human corneal epithelial cells after 30 min of culture in 160 μM pioglitazone. Hyperpure water (negative control), DMSO (negative control), and methyl acetate (positive control) were used. Cell viability was assessed by MTT assay. Each measurement was obtained using a microplate reader. The relative values are indicated. n = 6. Data represent mean ± SD. (K) A schematic diagram shows the binding of all-*trans*-RA to the retinoic acid receptor (RAR) and 9-*cis* RA to the retinoid X receptor (RXR), which then binds to the RA responsive sequence, such as retinoic acid response element (RARE) or retinoid X response element (RXRE), to induce the expression of the target gene. Liarozole inhibits the differentiation of all-*trans*-RA and suppresses RA metabolism. However, precisely how the RA metabolism functions in hiPSCs under vitamin A-free culture conditions has not yet been examined. (L) A schematic diagram showing that the PPAR/RXR complex binds to a PPRE composed of a direct repeat (DR) preferably spaced by 1 nt (DR1) with a consensus sequence of AGGTCA-n-AGGTCA. When RXR forms a dimer with PPARγ, the co-repressor inhibits binding to PPRE. 9-*cis* RA and pioglitazone promote the dissociation of co-repressors from the PPAR/RXR complex. (M–Q) Cell growth and viability assays of hiPSCs (201B7) in serum- and albumin-free medium after 48 hr of culture in the presence of 30 μM liarozole (M), 30 μM 13-*cis* RA (N), 30 μM 9-*cis* RA (O), 30 μM fenofibrate (P), and 30 μM bezafibrate (Q). The cells were stained for AP. Colony areas per well were measured. The relative values are indicated. n = 4. Data represent mean ± SD. **p < 0.01. (R) Cell growth and viability assays of hiPSCs (201B7) in serum- and albumin-free medium after 48 hr of culture in the presence of 1 μM atorvastatin and the absence or presence of 10 pmol/mL CYP3A4. The cells were stained for AP. Colony areas per well were measured. The relative values are indicated. n = 3. Data represent mean ± SD. *p < 0.05; **p < 0.01. (S) Cell growth and viability assays of hiPSCs (201B7) in serum-replacement-containing medium after 48 hr of culture in the presence of 10 μM atorvastatin, 30 μM liarozole, 25 μM pioglitazone, 20 μM silibin, and 40 μM chrysin. The cells were stained for AP. Colony areas per well were measured. The relative values are indicated. n = 4. Data represent mean ± SD. *p < 0.05; **p < 0.01. (T) Cell growth and viability assays of hiPSCs (201B7) in MEF-CM after 48 hr of culture in the presence of 30 μM liarozole, 25 μM pioglitazone, 20 μM silibin, and 40 μM chrysin. The cells were stained for AP. Colony areas per well were measured. The relative values are indicated. n = 4. Data represent mean ± SD. *p < 0.05.

consisting of catalytic subunits (p110α, p110β, and p110δ) and regulatory subunits (p85α, p55α, p50α, p85β, and p55γ). The catalytic subunit p110α is encoded by PIK3CA, and the regulatory subunit p85α is encoded by PIK3R1. The authors investigated the mRNA expression levels of PIK3CA and PIK3R1 by a qPCR method, by adding atorvastatin to the hiPSC medium after 24 hr (Figure S6). These results showed that atorvastatin significantly suppressed the PIK3CA and PIK3R1 mRNA expression levels of hiPSCs after 24 hr. RWD domain-containing protein 3 (RWDD3) enhances the sumoylation of several proteins including HIF1α and I-kappa-B, through direct interaction with UBC9.[43,44] Reduced expression of RWDD3 in hiPSCs causes suppression of the transcription activity of HIF1α. Atorvastatin increased the mRNA expression of HIF1α hydroxylase Egl 9 homolog 3 (EGLN3), an inhibitor of nuclear factor κB (NF-κB).[45,46] The insulin receptor (INSR) acts on the uptake of glucose into cells, and the very low-density lipoprotein receptor (VLDLR) acts on the uptake of LDL into cells. Atorvastatin strongly suppressed the INSR mRNA expression of hiPSCs and strongly promoted the VLDLR mRNA expression (Figures 4A and 4B). In addition, atorvastatin suppressed the mRNA expression of peroxisome proliferator-activated receptor (PPAR) δ and PPARα, a PPAR subtype (Figures 4A and 4B; Figure S6). PPARα is a ligand-activated transcription factor belonging to PPARγ and PPARδ. PPARδ reportedly reduces the oxidative stress of cancer cells and enhances the survival signaling response.[47] mRNA expression analyses revealed that atorvastatin is involved in the hypoxia response signal and HIF signal of hiPSCs.

### Atorvastatin Attenuates the Expression of Undifferentiated Markers

When atorvastatin 20 μM had been added to the serum- and albumin-free medium (Essential 8 medium) of hiPSCs for 6 hr, the margin of the colony changed to a rough form (Figure 4C, top panels). The expression of OCT3/4 and SOX2 was halved when the mRNA expression of an undifferentiated marker was examined. In addition, the expression of KLF4, C-MYC, NANOG, REX1, and DNMT3b sharply decreased (Figure 4C, lower panel). Certain metabolites of atorvastatin are known to have pharmacological effects.[48] When cytochrome P450 3A4 (CYP3A4) was added to atorvastatin-treated serum- and albumin-free medium, and atorvastatin is enzymatically differentiated, the undifferentiated hiPSC AP-positive colony area was significantly amplified. Experiments using CYP3A4 were conducted as a model of the possible drug metabolism *in vivo*. This indicates that non-metabolized atorvastatin has an inhibitory effect on hiPSCs (Figure 4R).

### HIF1α Has a Role in Maintaining the Survival of hiPSCs

Atorvastatin significantly reduced the expression of HIF1α, while other statins caused a non-significant reduction in gene expression (Figure 4D). The HIF1α inhibitors silibin (also called silybin or silibinin), the main component of silymarin[49–52] and chrysin[53] were added to the medium to investigate the effect of HIF1α on the suppression by atorvastatin of the viability of hiPSCs. On adding 20 μM silibin (Figure 4F) and 40 μM chrysin (Figure 4G) to serum- and albumin-free medium of hiPSCs for 48 hr, AP-staining-positive hiPSCs disappeared from the culture plate. Increased expression of HIF1α is characteristic of naive hiPSCs.[54] HIF1 reportedly plays an important role in the regulation of intracellular metabolism in the reprogramming of human pluripotent cells.[55]

### PPARγ Has a Role in Maintaining the Survival of hiPSCs

We next focused on the effect of PPARs on the survival activity of hiPSCs and studied the mechanism of action of atorvastatin. Fenofibrate (PPARα agonist) and bezafibrate (PPARδ agonist) did not affect the survival activity of hiPSCs (Figures 4P and 4Q). After adding pioglitazone, an agonist of PPARγ, to serum- and albumin-free medium at 25 μM, the survival activity of hiPSCs was significantly decreased, and colonies positive for AP staining disappeared from the inside of the plate after 48 hr (Figure 4H). This result shows that PPARγ[56] has a role in the nucleus of hiPSCs.

### Effect of Pioglitazone (PPARγ Agonist) on the Survival Inhibition of hiPSCs under Somatic Cell Culture Conditions

Human corneal epithelial cells and hiPSCs were mixed and cultured, and the effect of pioglitazone was investigated. Pioglitazone exerted a significant removal effect on hiPSCs at 3.2-fold the concentration of medium added compared with hiPSCs alone (Figure 4I, left panel). Under mixed culture conditions, when 80 μM pioglitazone was added to human corneal epithelial cells and hiPSCs in DMEM containing 10% FBS as a typical cell culture medium, the colony area of undifferentiated hiPSCs significantly decreased after 48 hr. Microscopic images after staining with AP showed colonies of undifferentiated hiPSCs stained in blue (Figure 4I, right panels). Overall, pioglitazone exerted a removal effect on hiPSCs, even under optimal culture conditions for human corneal epithelial cells. In the three-dimensional culture test using human corneal epithelial cells, the safety of the ingredients of eye drops was investigated. Pioglitazone at 160 μM did not show any abnormalities as a component of eye drops (Figure 4J). This result indicates that the corneal epithelial cells, which are mature cells, do not promote the survival activity of hiPSCs.

We examined the presence (or absence) of factors that interfere with the effects of five drugs (atorvastatin, liarozole, pioglitazone, silibin, and chrysin) that were shown to suppress the survival of undifferentiated hiPSCs in this study. If these drugs influence the metabolism of hiPSCs, it is possible that the suppression of hiPSC survival may be attenuated depending on the components of the medium. Even when KnockOut Serum Replacement (KSR), a nutritional supplement, was added to the hiPSC medium, the five drugs significantly inhibited the survival of hiPSCs. These results indicate that the five drugs have a sufficient effect, even when common iPSC medium is used, or differentiation induction medium with serum replacement or the addition of human albumin is used (Figure 4S). Next, we examined the effects of four types of drugs (liarozole, pioglitazone, silibin, and chrysin) that suppressed the survival of hiPSCs with MEF conditional medium (MEF-CM) (culture supernatant of MEF cultured overnight) supplemented with KSR. As a result, due to the influence of the components secreted by MEF, the effect of the four drugs with regard to the suppression of hiPSC survival was remarkably

decreased, and a significant effect was observed only with chrysin. These results indicate that MEF-CM has an extremely strong effect with regard to enhancing the survival of hiPSCs. Thus, the drugs that we found to inhibit the survival of hiPSCs cannot exert their effects in culturing experiments with human or mouse fetal cells (Figure 4T).

### Sustained Metabolic Elimination of Cellular RA Maintains the Survival of hiPSCs

PPARs and retinoic acid (RA) receptor (RAR) form a heterodimer with the retinoid X receptor (RXR), which binds to the response sequence of the promoter region (peroxisome proliferator response sequence and RA response sequence) to induce expression of each target gene.[57,58] Surprisingly, the addition of 30 μM liarozole (inhibitor of CYP26 that acts on RA metabolism) in vitamin A-free and serum- and albumin-free medium significantly reduced the survival activity of hiPSCs (Figure 4M). This result shows that factors related to cellular RA metabolism may be involved in the survival maintenance in undifferentiated hiPSCs.

### RXR Is a Nuclear Factor that Plays a Role in Maintaining the Survival of hiPSCs

Next, the RAR was activated to investigate the survival activity of undifferentiated hiPSCs. 13-*cis* RA of the precursor of all-*trans* RA, a ligand of nuclear receptor RAR, was added to the medium at 30 μM (Figure 4N). In addition, 9-*cis* RA, a ligand of the nuclear receptor RXR, was added at a concentration of 30 μM to the medium (Figure 4O). Liarozole appeared to enhance the activity of nuclear receptor RAR and RXR of hiPSCs by inhibiting RA metabolism[59] (Figure 4K). 13-*cis* RA was converted to all-*trans* RA in the body. All-*trans* RA was then converted to 9-*cis* RA, and both 13-*cis* RA and 9-*cis* RA shrank AP-positive hiPSC colonies. These findings suggest that RXR plays an extremely important role in maintaining the survival activity of hiPSCs.

### The HIF1α-PPARγ Axis Maintains the Survival of hiPSCs

RXR forms heterodimers with PPARγ.[60,61] Nuclear receptor co-repressor binds to PPARγ-RXR heterodimer, and the transcription activity is suppressed. However, pioglitazone, which is a ligand of PPARγ, or 9-*cis* RA, which is a ligand of RXR, dissociates the co-repressor bound to the dimer. The co-activator then combines to form peroxisome proliferator response elements (PPREs) (AGGTCA-n-AGGTCA direct repeat structure), which are subsequently transferred (Figure 4L). Previous studies have reported that RXR-PPARγ promotes p27kip expression,[62,63] which is consistent with our results using hiPSCs (Figure S3H). From the results of this study, we conclude that the formation of the HIF1α-PPARγ axis and the related RXR-PPARγ heterodimer with no bound ligand have an important role in maintaining the survival activity of hiPSCs.

## DISCUSSION

Several strategies have been suggested for the removal of residual pluripotent cells from differentiated cultures: cell sorting of hPSCs using immunological targeting based on specific antigens for pluripotency;[64–67] elimination using cytotoxic antibodies;[68,69] genetic manipulation of potential tumor progression genes in hPSCs;[70,71] insertion of genes to induce cell death after a specific signal,[71–73] identification of improved ways to separate hPSCs from differentiated cells in culture;[74–76] synthesis of peptides to induce cell death by binding on the surface AP on hiPSCs;[77] elimination of residual hiPSCs targeting CD30;[78] and identification of compounds and/or treatments that specifically kill pluripotent cells in cultures after induced cell differentiation.[10,12,64,79,80] Although various methods have been developed to remove undifferentiated hPSCs, in general, they are difficult to combine with clinical therapeutic methods, and whether or not they completely remove undifferentiated cells is often unclear. At present, clinical trials using differentiated human embryonic stem cells (ESCs) strive to achieve 100% differentiation through the use of agents such as bone morphogenetic factors (BMPs) from the transforming growth factor β (TGF-β) family of ligands. However, even with long-term differentiation protocols, undifferentiated cells may still be present and be capable of inducing tumor formation after injection into an animal host.[81–84] There is, therefore, considerable interest in the development of consistently reliable methods to remove or kill undifferentiated stem cells following the induction of cell differentiation.

Initially, we had to develop a way to identify survival inhibitors of hiPSCs. The morphology of hiPSC colonies is a significant indicator of important characteristics of cell culture, such as cell adhesion, cell death, cell proliferation, and the undifferentiated state of adherent hiPSCs.[85,86] We then applied electrochemical impedance to investigate agents that reduce the survival of pluripotent stem cells in medical products. The drug concentration of statins used here, 10 μM, was calculated as approximately 150 mg/day given to patients (assuming a patient body weight of 75 kg and body fluid volume of 60%). The Food and Drug Administration (FDA) recommended a maximum dosage of 80 mg/day simvastatin.[16,87,88] Therefore, with the current dosage recommended by the FDA, it is difficult to completely eliminate hiPSCs *in vivo* by killing undifferentiated hiPSCs in the body through the ingestion of atorvastatin. In the present study, atorvastatin (concentrations of 1–20 μM) showed a cell survival-inhibiting effect on hiPSCs in the culture medium (exposure time: 6–96 hr). This cell survival-inhibiting effect involved a wide range of cellular reactions, including cytotoxicity. It is known that serum contains various antioxidants. Because silibin and chrysin are antioxidants, the antioxidant activity of silibin and chrysin, especially under serum-free culture conditions, may have a significant influence on the cell survival of hiPSCs. Thus, when serum-free medium is used, the survival inhibitory effect of silibin and chrysin on hiPSCs would likely be influenced by a strong antioxidant mechanism that differs from the mechanism of action exhibited by atorvastatin. However, in this paper, we showed that HIF1α inhibitors could be used in internal medicine to eliminate residual hiPSCs inside a patient's body.

Atorvastatin eliminates hiPSCs mainly via the inhibition of PI3K/AKT signaling. This signaling is responsible for integrin- and growth factor-mediated signals using nutrients such as glucose and amino acids.[89] The cell attachment levels in hiPSC cultures affect the cell

viability and are mediated through the Rho-ROCK signaling pathway.[24,32] Basically, statins prevent the geranylgeranylation and farnesylation of small GTPases (e.g., geranylgeranylation of RhoA)[90] and inhibit integrin signaling, which can induce the death of progenitor cells.[91] Indeed, in addition to atorvastatin, fluvastatin, lovastatin, mevastatin, and simvastatin were also found to have a weak inhibitory effect on hiPSCs (Figures 2C and 2D). However, we showed that the ROCK inhibitor (Y-27632) could not rescue hiPSCs from the survival-suppressing effect of atorvastatin (Figure 2F) and fluvastatin (Figure 2I). With regard to the interpretation of this result, Schmidmaier et al.[92] reported that the suppression of HMG-CoA signaling through farnesylated small G proteins (e.g., Ras) and geranylgeranylated small G proteins (e.g., Rho; inhibited by Y-27632) is divided into two routes; our results suggested the possibility that atorvastatin might affect both the Rho and Ras pathways in hiPSCs. A previous study found that atorvastatin caused the level of AKT activity to fall below the threshold necessary to maintain other types of cell survival.[93] Different isoforms of AKT may therefore have overlapping roles,[94,95] and LY-294002 and atorvastatin might act on different AKT isoforms. The first generation of AKT inhibitors, such as LY-294002, had serious side effects and high toxicity.[96] Atorvastatin may therefore represent a new AKT inhibitor for hiPSCs. We believe that atorvastatin acts to reduce the hiPSC survival and induces the loss of pluripotency via the PI3K/AKT pathway by stimulating integrin and growth factor signaling. The authors added atorvastatin (20 $\mu$M $\times$ 24 hr) to the culture medium of hiPSCs and measured the mRNA expression level using a qPCR. It was revealed that atorvastatin suppressed the mRNA expression of PIK3CA and PIK3R1, which is a constituent of PI3K/AKT (Figure S6). This result implies that atorvastatin and fluvastatin have an indirect effect of suppressing the PI3K/AKT signaling of hiPSCs, which is unlike other statins. However, the possibility that the results were observed because of the cytopathic effects of atorvastatin and fluvastatin cannot be ruled out. $H_2O_2$ stress, a common cause of cytotoxicity, affects many signaling pathways, including the HIFs, PI3K, NF-κB, and MAPK. It has also been reported that reactive oxygen species (ROS), a representative antioxidant factor, control the self-renewal and differentiation of stem cells.[97] Thus, we recognize that it is necessary to carefully consider the relationship between the results of this study and the oxidative stress response.

Previous studies have shown that ESCs and iPSCs have only a few mitochondria with immature morphologies[98–103] and, upon differentiation, acquire more mitochondria with mature features, such as fully developed cristae, a denser matrix, and increased oxidative capacity.[101,103] Therefore, the mechanism underlying cellular metabolism under hypoxic conditions is enhanced in hiPSCs. Furthermore, it has been reported that HIFs promote the expression of hiPSC marker genes.[104] It is also known that HIF1α[105] is involved in the PI3K signaling pathway.[106] Therefore, we inferred that atorvastatin inhibited the PI3K/AKT-HIF1α signaling of hiPSCs.

The existence of an HIF1α-PPARγ axis has been debated. In cardiomyocytes and cancer cells, HIF1α has been reported to activate PPARγ.[107,108] In pulmonary vascular cells, PPARγ is reportedly subject to HIF1-dependent and HIF1-independent adjustment.[109] In addition, negative feedback in which PPARγ suppresses HIF1α has been reported.[110] A relationship has also been observed between the cell cycle and PPARγ in nerve cells.[111] In this paper, we indicated five agents (atorvastatin, liarozole, pioglitazone, silibin, and chrysin) that attenuate the HIF1α-PPAR axis in hiPSCs. However, we also admit that there is room for further discussion of this hypothesis. We agree that the data to prove the hypothesis regarding the HIF1A/PPAR axis signaling pathway of hiPSCs is inadequate. We conducted additional experiments based on the interpretation of the experimental results, which were speculative. First, the mRNA expression of an undifferentiated marker expressed in hiPSCs was examined by a qPCR after the addition of a drug (atorvastatin, LY 294002, silibin, chrysin, pioglitazone, liarozole) that had the effect of suppressing the survival of the hiPSCs used in this experiment (Figures S7A and S7B). The expression of an undifferentiated marker of hiPSCs was affected by various reagents; however, while there was overall variation, a trend toward inhibition was observed in markers other than C-MYC. This result indicates that although the selected drug did not cause the action on the perfectly matched hiPSC signaling pathway, it caused the inhibition of the undifferentiated marker on similar hiPSCs. The similar action of various drugs against hiPSCs suggested the presence of the HIFα-PPAR axis. It has already been shown that atorvastatin reduces the HIFα mRNA expression of hiPSCs (Figure 4D). Thus, the authors examined changes in the mRNA expression levels of PI3K/AKT (PIK3CA and PIK3R1), RWDD3, EGLN3, PPARα, PPARδ, and PPARγ (described in the hypothetical diagram described in Figure 4E)[43,45,63,108,112–117] using a qPCR (Figure S6). This revealed that atorvastatin significantly reduced the mRNA expression levels of hiPSC PIK3CA, PIK3R1, RWDD3, EGLN3, PPARα, and PPARδ after 24 hr. This result shows that the presence of the HIF1α-PPAR axis maintains the survival activity of hiPSCs. However, in the experimental data (Figure 4B), when mRNA was sampled at 6 hr after the administration of atorvastatin, the mRNA expression level of EGLN3 increased. We increased the number of samples (n = 3) and performed a replacement test. Then the EGLN3 mRNA expression of hiPSCs increased at 6 hr after the administration of atorvastatin (data not shown). This result was different from the results obtained at 24 hr after the administration of atorvastatin (Figure S6). More detailed research on the influence of hiPSCs on NF-κB signaling following the administration of atorvastatin should be performed in the future.

The survival activity inhibitory effect of these five drugs on hiPSCs was highest under serum- and albumin-free medium conditions, and the effect was attenuated in serum or serum replacement (KSR). This result shows that it is difficult to completely inhibit the survival of undifferentiated hiPSCs *in vivo* with drugs at the usual dosage (Figure 4S). MEF-CM made with serum replacement such as KSR markedly reduced the effect of the above drugs on inhibiting the HIF1α-PPAR axis (Figure 4T). One reason for this is considered to be that MEF feeder cells and MEF-CM strongly activate the PI3K/AKT signal.[112] However, there are reports that HIF1α is not required

for the establishment of hiPSCs.[118] This indicates that the survival activity of hiPSCs may be maintained by both HIF1α-dependent and HIF1α-independent signals when MEF feeder cells are used.

In conclusion, the HIF1α-PPAR axis contributes to the maintenance of survival activity of major hiPSCs under serum- and albumin-free culture conditions. Therefore, suppression of the survival activity of hiPSCs cultured in serum- and albumin-free medium supplemented with atorvastatin enhances the safety for producing human cell-based medicine. The inhibitory effect of atorvastatin on hiPSC survival activity may substantially reduce the potential risk for carcinogenesis after transplanting the product to patients.

## MATERIALS AND METHODS

### Reagents

DMEM, DMEM and nutrient mixture F-12 Ham 1:1 (DMEM-F12), FBS, non-essential amino acid solution (100×), 2-mercaptoethanol, Tris-buffered saline, Trizma hydrochloride solution, and methyl acetate were obtained from Sigma-Aldrich (St. Louis, MO, USA). KnockOut Serum Replacement (KSR), L-glutamine, collagenase type IV, DMSO, and Essential 8 medium were obtained from Life Technologies (Carlsbad, CA, USA). Calcium chloride, gelatin, mitomycin C, formaldehyde solution, Y-27632, and LY-294002 were purchased from WAKO (Osaka, Japan). Recombinant human basic fibroblast growth factor (bFGF) was obtained from ReproCELL (Kanagawa, Japan). D-PBS(−) was obtained from Nacalai Tesque (Kyoto, Japan). Trypsin and Growth Factor Reduced BD Matrigel were purchased from Becton Dickinson (Franklin Lakes, NJ, USA). The PSdif-Cardio and Cardiomyocyte Differentiation kit was obtained from StemRD (Burlingame, CA, USA). The PSC Cardiomyocyte Differentiation Kit was obtained from Thermo Fisher Scientific (Kanagawa, Japan). Recombinant human MFG-E8 and a Human Pluripotent Stem Cell Functional Identification Kit (SC027B) were purchased from R&D Systems (Minneapolis, MN, USA). Atorvastatin, fluvastatin, lovastatin, mevastatin, simvastatin, phlorizin hydrate, silibin (also called silybin or silibinin), 13-*cis*-retinoic acid, bezafibrate, and fenofibrate were purchased from Tokyo Chemical Industry (Tokyo, Japan). 9-*cis*-Retinoic acid was purchased from LKT Laboratories (St. Paul, MN, USA). Chrysin and CYP26A1 (F27 P6 A1) were obtained from Santa Cruz Biotechnology (Santa Cruz, CA, USA). Liarozole hydrochloride was purchased from Tocris Bioscience (Bristol, UK). Anti-troponin T, cardiac isoform Ab-1 (Clone 13-11) antibody was obtained from Thermo Fisher Scientific (Kanagawa, Japan). Goat anti-mouse immunoglobulin M (IgM) mu chain (Alexa Fluor 488) was obtained from Abcam (Cambridge, UK), and goat anti-rabbit IgG (H+L) and HiLyte Fluor 488 from AnaSpec (Fremont, CA, USA). Anti-PI3-kinase (p85α) monoclonal antibody (mAb) was obtained from Medical & Biological Laboratories (Nagoya, Japan). Purified Mouse Anti-PI3-Kinase p110α antibody was obtained from Becton, Dickinson (Franklin Lakes, NJ, USA). β-Actin (C4) antibody was obtained from Santa Cruz Biotechnology (Dallas, TX, USA). Anti-mouse IgG, horseradish peroxidase (HRP)-linked antibody was from Cell Signaling Technology (Boston, MA, USA). Blue Alkaline Phosphatase Substrate Kit was obtained from Vector Laboratories (Cambridgeshire, UK). Permeabilization buffer (10×) was from eBioscience (Vienna, Austria). Cell characterization kits were obtained from System Biosciences (Mountain View, CA, USA). Plastic dishes were obtained from TPP (Trasadingen, Switzerland). Other materials used were of the highest commercial grade.

### Maintenance Culture of hiPSCs

The hiPSC lines 201B7, 253G1, and 409B2 were established by Shinya Yamanaka (Kyoto University) and were obtained from RIKEN BioResource Center (Ibaraki, Japan). The cells were cultured on a feeder layer of MEF cells (Oriental Yeast) that had been inactivated with 10 μg/mL mitomycin C and seeded at $1.5 \times 10^5$ cells per 10-cm plate. The cells were cultured in DMEM/F12 supplemented with 10% FBS, 2 mM L-glutamine, 0.1 mM nonessential amino acids, 5 ng/mL recombinant human bFGF, and 0.1 mM 2-mercaptoethanol under 3% $CO_2$. For passaging, hiPSCs colonies were treated with 0.25% trypsin and 0.1 mg/mL collagenase IV in PBS containing 20% KSR and 1 mM $CaCl_2$ at 37°C for 5 min, followed by tapping the cultures and flushing them with a pipette. Two volumes of culture medium were added, and the detached hiPSCs clumps were broken into smaller pieces by gentle pipetting. The passages were performed using a 1:3 split ratio. For storage, hiPSCs colonies were placed into human ESC/iPSC freezing medium according to the manufacturer's instructions (ReproCELL). To prepare seeding hiPSCs for each cell assay, the hiPSCs were first detached from the feeder layer and partially dissociated as described for maintenance passage. Next, the contaminating MEF cells were removed by incubating the cell suspension on a gelatin-coated plate at 37°C for 2 hr in Essential 8 medium with 10 μM Y-27632 to ensure high purity of the hiPSCs. The differentiated hiPSCs were cultured for 10 days, minus passage on a feeder layer of MEF cells. The day on which hiPSCs were seeded to start the dissociation culture was defined as day 0. Differentiated hiPSCs were prepared using a medium that had been preserved for 6 months or longer and whose protein components had been degraded. Differentiation was selected by roughing of the colony form as an index under microscopic observation.

### Cell Differentiation Assays

Myocardial cells were induced to differentiate from hiPSCs cultured to confluence in 96-well plates in Essential 8 medium (normal or MEF-CM) using a PSdif-Cardio Cardiomyocyte Differentiation kit (StemRD) and a PSC Cardiomyocyte Differentiation Kit (Thermo Fisher Scientific) according to the manufacturer's instructions. These myocardial cells differentiation induction kits were manufactured according to the methods of previous studies.[119,120] The wells were coated or not coated with Growth Factor Reduced BD Matrigel. iPSCs, mesendoderm, cardiac mesoderm, myocardial precursor cells, and myocardial cells were regulated according to the manufacturer's instructions. Ectoderm, mesoderm, and endoderm were induced to differentiate from hiPSCs cultured to confluence in 96-well plates using a Human Pluripotent Stem Cell Functional Identification Kit (SC027B; StemRD) according to the manufacturer's instructions.

### Cell Proliferation Assays

Cell proliferation was measured using a Cell Counting Kit-8, Cell Counting Kit (Dojindo, Kumamoto, Japan) and Cell Count Reagent

SF and MTT Cell Count Kit (Nacalai Tesque) according to the manufacturers' instructions. In brief, the cells were seeded into 96-well plates coated with 1.8 μL per $cm^2$ of Growth Factor Reduced BD Matrigel. Each well received 2.5 × $10^5$ cells/mL Essential 8 medium. Absorbance was measured with a microplate reader at a wavelength of 450–570 nm. A neutral red uptake assay was performed using neutral red (Nacalai Tesque). In brief, 3 hr before the end of the incubation period, the medium was aspirated from each well. Immediately, a working neutral red solution (0.05% of the neutral red stock in cell culture medium without serum) was added to each well, and the cells were incubated for 3 hr. At the end of the incubation period, the neutral red solution was removed, and the wells were washed with warm PBS (37°C). Subsequently, a fixative solution (150 μL) was added to each well, and absorbance was measured at 450–570 nm on a plate reader.

### Cell Death Assays

hiPSC death was measured using the Cytotoxicity LDH Assay kit (Dojindo) according to the manufacturer's instructions. In brief, the cells were seeded into 96-well plates coated with 1.8 μL per $cm^2$ Growth Factor Reduced BD Matrigel. Each well received 2.5 × $10^5$ cells/mL Essential 8 medium. Absorbance was measured with a microplate reader at a wavelength of 490 nm. Myocardial cell death was assessed using a Live/Dead Cell Staining kit according to the manufacturer's instructions (PromoCell, Heidelberg, Germany). Images were recorded using a DMI 4000B fluorescence microscope (Leica, Wetzlar, Germany).

### Cytotoxicity Assay

Human corneal epithelial cells were obtained from the KURABO Bio-Medical department (Osaka, Japan). CM was prepared by culturing human corneal epithelial cells (3 × $10^6$ cells) with 10 mL of 10% FBS-containing DMEM medium for 24 hr. The hiPSCs (5 × $10^3$ cells/mL/24 wells) were seeded in a colony state and co-cultured against human corneal epithelial cells (1 × $10^4$ cells/mL/24 wells). The human corneal three-dimensional model OCL-200EIT[121,122] (MatTek Corporation, Ashland, MA, USA) was used according to the manufacturer's instructions.

### Measuring Electrochemical Impedance

Impedance measurement was performed with an xCELLigence RTCA DP analyzer (ACEA Biosciences, San Diego, CA, USA), and readings were taken every 5 min. Single-purpose measurement 16-well plates were coated with 10 μL per well of Growth Factor Reduced BD Matrigel or 1 μg per well of iMatrix-511 (Nippi, Tokyo, Japan). Each well was seeded with 2.5 × $10^5$ hiPSCs/mL or confluent developing myocardial cells and cultured in appropriate medium: Essential 8 medium or MEF-CM Essential 8 medium. An experimental compound was also added: 2.5 μg/mL MFG-E8, 10 μM atorvastatin, 10 μM fluvastatin, 10 μM lovastatin, 10 μM mevastatin, or 10 μM simvastatin.

### Real-Time PCR and RT-PCR

Cells were cultured in 96-well plates in Essential 8 medium to approximately 80% confluence. RNA was prepared using a SuperPrep Cell Lysis and RT kit for qPCR according to the manufacturer's instructions (Toyobo, Osaka, Japan). Real-time PCR analyses were performed using a StepOnePlus system (Life Technologies) or a LightCycler 96 Real-Time PCR system (Roche, Basel, Switzerland). The Thunderbird SYBR qPCR Mix (Toyobo) and FastStart Essential DNA Green Master (Roche) were used according to the manufacturer's instructions (Toyobo). An RT-PCR analysis was performed using a GeneAtlas 322 thermal cycler (Astec, Fukuoka, Japan). Images were recorded using an Atto AE-6932GXCF system (Atto, Tokyo, Japan). The Quick Taq HS DyeMix was used according to the manufacturer's instructions (Toyobo). For the design of primers other than the primers cited in other papers, the gene names were retrieved from the US National Library of Medicine NIH website (https://www.ncbi.nlm.nih.gov/pubmed/). The primers were designed using the Primer 3 Plus application (http://www.bioinformatics.nl/cgi-bin/primer3plus/primer3plus.cgi). Human HIFα-1 primers (sc-35561-PR) were obtained from Santa Cruz Biotechnology and were performed using primers listed in Table 1. The other primers used for PCR have been described previously[1,123,124] and were as follows:

human OCT3/4 forward, 5′-GACAGGGGGAGGGGAGGAGCTAGG-3′,

human OCT3/4 reverse, 5′-CTTCCCTCCAACCAGTTGCCCCAAAC-3′,

human SOX2 forward, 5′-GGGAAATGGGAGGGGTGCAAAAGAGG-3′,

human SOX2 reverse, 5′-TTGCGTGAGTGTGGATGGGATTGGTG-3′,

human KLF4 forward, 5′-TGATTGTAGTGCTTTCTGGCTGGGCTCC-3′,

human KLF4 reverse, 5′-ACGATCGTGGCCCCGGAAAAGGACC-3′,

human c-MYC forward, 5′-GCGTCCTGGGAAGGGAGATCCGGAGC-3′,

human c-MYC reverse, 5′-TTGAGGGGCATCGTCGCGGGAGGCTG-3′,

human NANOG forward, 5′-CAGCCCCGATTCTTCCACCAGTCCC-3′,

human NANOG reverse, 5′-CGGAAGATTCCCAGTCGGGTTCACC-3′,

human GDF3 forward, 5′-CTTATGCTACGTAAAGGAGCTGGG-3′,

human GDF3 reverse, 5′-GTGCCAACCCAGGTCCCGGAAGTT-3′,

human REX1 forward, 5′-CAGATCCTAAACAGCTCGCAGAAT-3′,

human REX1 reverse, 5′-GCGTACGCAAATTAAAGTCCAGA-3′,

**Table 1. Primer Sequences for Real-Time qPCR**

| Genes | Accession Number | Sequences (5′→3′) | Length (bp) |
|---|---|---|---|
| Human T brachyury | NM_001270484 | Forward: GCTGAACTCCTTGCATAAGTATGAG<br>Reverse: CATCTCTTTGTGATCACTTCTTTCC | 211 |
| Human KDR | NM_002253 | Forward: AAGTAGAGTTCGTTGTGCTGTTTCT<br>Reverse: TCAGGACAGATATGAGGGTATTCAT | 248 |
| Human ISL1 | NM_002202 | Forward: ATATGGTAGCAACACTGTGAAGACA<br>Reverse: CCATTCAGATTAGGATGGACTAGAA | 234 |
| Human NKX2.5 | NM_001166175 | Forward: GAAATTTTAAGTCACCGTCTGTCTC<br>Reverse: AGTAATGGTAAGGGATCCTCGTG | 221 |
| Human TNNT2 | NM_000364 | Forward: AAAGAAGAAGAAGATTCTGGCTGAG<br>Reverse: GATCTCATATTTCTGCTGCTTGAAC | 166 |
| Human CCND1 | NM_053056 | Forward: CCCCTTGATTTAAACACACAGATAC<br>Reverse: AGGTTGAGTACCCTAATTTTCCTTG | 235 |
| Human INSR | NM_000208 | Forward: CTGGATCCAATCTCAGTGTCTAACT<br>Reverse: GAATCCTCATACTCACTCTGGTTGT | 239 |
| Human PPARA | NM_001001928 | Forward: ATCCTCTCTCCAACTTCATACCTCT<br>Reverse: CAATACATGTGGTCAGTTCAGTCTC | 220 |
| Human PPARG | NM_001330615 | Forward: GATATCAAGCCCTTCACTACTGTTG<br>Reverse: TCTCAGAATAATAAGGTGGAGATGC | 175 |
| Human PPARD | NM_001171818 | Forward: TCCTCCTTTCTTATTCTGTGAGATG<br>Reverse: AGAGAGCTTAGTGTTTCTTTGGATG | 210 |
| Human EGLN3 | NM_001308103 | Forward: GTGTGTGGTACTTCATGTTTTCTTG<br>Reverse: GCTCCTAGGCTCTTCTCTTGATAGT | 165 |
| Human PIK3CA | NM_006218 | Forward: CTTTAAGAGAAGGCTGAAAGTTGTG<br>Reverse: CAG CTGTATTATTCTGTCACCAAGA | 152 |
| Human PIK3R1 | NM_001242466 | Forward: CCCAGTGTAGCATCCTAAAGATAAA<br>Reverse: CAACAACTACTGTGGATCTGTTTTG | 247 |
| Human RWDD3 | NM_001128142 | Forward: GATTTATGGATGCGGATATACCTCT<br>Reverse: CTCTCTGCTTGCTCAAGTAAATTCT | 151 |

human DNMT3b forward, 5′-TGCTGCTCACAGGGCCCGATACTTC-3′,

human DNMT3b reverse, 5′-TCCTTTCGAGCTCAGTGCACCACAAAAC-3′,

human LIN28 forward, 5′-AGCCATATGGTAGCCTCATGTCCGC-3′,

human LIN28 reverse, 5′-TCAATTCTGTGCCTCCGGGAGCAGGGTAGG-3′,

human RhoA forward, 5′-CATCCGGAAGAAACTGGT-3′,

human RhoA reverse, 5′-TCCCACAAAGCCAACTC-3′,

human Cyclin D1 forward, 5′-CACACGGACTACAGGGGAGT-3′,

human Cyclin D1 reverse, 5′-CACAGGACCTCGTGTTCCAT-3′,

human Cyclin D1 forward, 5′-CCCCTTGATTTA AAC ACACAGATAC-3′,

human Cyclin D1 reverse, 5′-AGGTTGAGTACCCTAATTTTCCTTG-3′,

human p21cip1 forward, 5′-CACCGAGACACCACTGGAGG-3′,

human p21cip1 reverse, 5′-GAGAAGATCAGCCGGCGTTT-3′,

human p65 forward, 5′-GGGGACTACCACCTGAATG-3′,

human p65 reverse, 5′-GGGCACGATTGTCAAAGAT-3′,

human p27kip1 forward, 5′-TAATTGGGGCTCCGGCTAACT-3′,

human p27kip1 reverse, 5′-TTGCAGGTCGCTTCCTTATTC-3′,

human GAPDH forward, 5′-ACCACAGTCCATGCCATCAC-3′,

human GAPDH reverse, 5′-TCCACCACCCTGTTGCTGTA-3′,

human β-actin forward, 5′-CAACCGCGAGAAGATGAC-3′, and

human β-actin reverse, 5′-AGGAAGGCTGGAAGAGTG-3′.

The primers used for RT-PCR were as follows:

human OCT3/4 forward, 5′-GACAGGGGGAGGGGAGGAGCTAGG-3′,

human OCT3/4 reverse, 5′-CTTCCCTCCAACCAGTTGCCCCAAAC-3′,

human SOX2 forward, 5′-GGGAAATGGGAGGGGTGCAAAAGAGG-3′,

human SOX2 reverse, 5′-TTGCGTGAGTGTGGATGGGATTGGTG-3′,

human KLF4 forward, 5′-GATTACGCGGGCTGCGGCAAAACCTACACA-3′,

human KLF4 reverse, 5′-TGATTGTAGTGCTTTCTGGCTGGGCTCC-3′,

human c-MYC forward, 5′-GCGTCCTGGGAAGGGAGATCCGGAGC-3′,

human c-MYC reverse, 5′-TTGAGGGGCATCGTCGCGGGAGGCTG-3′,

human NANOG forward, 5′-CAGCCCCGATTCTTCCACCAGTCCC-3′,

human NANOG reverse, 5′-CGGAAGATTCCCAGTCGGGTTCACC-3′,

human GDF3 forward, 5′-CTTATGCTACGTAAAGGAGCTGGG-3′,

human GDF3 reverse, 5′-GTGCCAACCCAGGTCCCGGAAGTT-3′,

human REX1 forward, 5′-CAGATCCTAAACAGCTCGCAGAAT-3′,

human REX1 reverse, 5′-GCGTACGCAAATTAAAGTCCAGA-3′,

human SAL4f forward, 5′-AAACCCCAGCACATCAACTC-3′,

human SAL4f reverse, 5′-GTCATTCCCTGGGTGGTTC-3′,

human DNMT3b forward, 5′-TGCTGCTCACAGGGCCCGATACTTC-3′,

human DNMT3b reverse, 5′-TCCTTTCGAGCTCAGTGCACCACAAAAC-3′,

human Nkx2.5 forward, 5′-GCGATTATGCAGCGTGCAATGAGT-3′,

human Nkx2.5 reverse, 5′-AACATAAATACGGGTGGGTGCGTG-3′,

human Troponin T forward, 5′-TTCACCAAAGATCTGCTCCTCGCT-3′,

human Troponin T reverse, 5′-TTATTACTGGTGTGGAGTGGGTGTGG-3′,

human β-actin forward, 5′-CAACCGCGAGAAGATGAC-3′,

human β-actin reverse, 5′-AGGAAGGCTGGAAGAGTG-3′,

human β-actin forward, 5′-TGGCACCCAGCACAATGAA-3′, and

human β-actin reverse, 5′-CTAAGTCATAGTCCGCCTAGAAGCA-3′.

### qPCR Array

For the mRNA expression analysis, a GeneQuery Human Hypoxia Response qPCR Array Kit (GQH-HPX) and Human HIF1 Signaling Response qPCR Array Kit (GQH-HFT) (ScienCell, Carlsbad, CA, USA) were used. A qPCR array (GQH-HPX) was designed to facilitate the gene expression profiling of 88 key genes involved in the cellular response to low-oxygen conditions. Brief examples of how the included genes may be grouped according to response type are as follows: cellular metabolism: ENO1, HK1, PFKFB3, PGK1, and SLC2A1; inflammation: CXCL8, IL-1A, CCL2, IL-6, and THBD; vasorelaxation: AHSP and PTGIS; cell death or proliferation: BNIP3L, NOS3, PIM1, and EPO; and angiogenesis: KDR, PDGFB, PLAU, TEK, and FLT1. A qPCR array (GQH-HFT) was designed to facilitate gene expression profiling of 88 key genes involved in HIF1 downstream signaling upon hypoxia-induced activation. HIF1 activity is a major component of the human response to hypoxia. Brief examples of how the included HIF1 downstream genes may be grouped according to the regulatory functions are as follows: cell cycling: DHFR, MAP3K5, POLE2, PRC1, and SKP2; extracellular matrix remodeling: COL1A1, COL5A1, LOX, P4HA1, and PLOD1; angiogenesis: HMOX1, MMP2, VEGFA, ANGPTL4, and PGF; and metabolism: PKM, STC2, DHX35, DDX11, and ERO1A.

### Immunofluorescence Staining Analysis

Immunofluorescence staining was performed using the human ES/iPS Cell Characterization kit (System Biosciences). Permeabilization buffer (10×; eBioscience, Vienna, Austria) was used instead of 0.1% Triton X-100 in PBS. Immunofluorescence staining was achieved using specific antibodies for troponin T, cardiac isoform Ab-1 (Clone 13-11; Thermo Fisher Scientific, Kanagawa, Japan), goat anti-mouse IgM mu chain (Alexa Fluor 488; Abcam), and goat anti-rabbit IgG (H+L) (HiLyte Fluor 488; AnaSpec). Images were recorded using a DMI 4000B fluorescence microscope (Leica).

### Western Blot Analysis

Cells were cultured in 150-mm dishes in Essential 8 medium to ∼80% confluence. Western blot analysis was performed using EzRIPA Lysis kit, PAGE Ace Twin, myPower II 300, HorizeBLOT 2M-R, EzApply, EzStandard PrestainBlue, c-PAGEL 10%, EzRun, P plus membranes, Filter paper, EzFastBlot, EzBlock Chemi, EzTBS, EzWeatBlue, and EzWestLumi One according to the manufacturer's instructions (ATTO, Tokyo, Japan). Blots were probed using specific antibodies for PI3-Kinase p85α (Monoclonal mouse Anti-PI3-kinase p85α; MD-06-3), PI3-Kinase p110α (Purified Mouse Anti-PI3-Kinase p110α; BD 611398) or β-Actin (C4) (Santa Cruz Biotechnology; sc-47778), and anti-mouse IgG, HRP-linked antibody from Cell Signaling Technology (7076S).

### AP Staining Analysis

AP staining was performed using a Blue Alkaline Phosphatase Substrate kit according to the manufacturer's instructions (Vector Laboratories).

### Animal Care

All experimental protocols were in accordance with the guidelines for the care and use of laboratory animals set by the Graduate School of the Institute of Health Biosciences, Tokushima University (Tokushima, Japan). The experimental protocol was approved by the Committee on Animal Experiments of the Tokushima University (permit number 14048). C.B-17 severe combined immunodeficiency (SCID) (CB17/Icr-Prkdcscid/Cr1Cr1j) male mice (8 weeks old; Charles River Laboratories Japan, Yokohama, Japan) were maintained under controlled temperature (23°C ± 2°C) and light conditions (lights on from 08:30–20:30), and fed standard rodent chow pellets (Oriental Yeast, Tokyo, Japan) with water *ad libitum*. All efforts were made to minimize the suffering of the animals.

### Teratoma Formation Assay

Immunodeficient mice (C.B-17 SCID) at 8 weeks of age were used for teratoma formation assays. hiPSCs were cultured with the medium with each reagent for 48 hr before being injected into mice. In brief, the teratoma formation mouse model was established by anesthetizing recipient mice with isoflurane inhalation (WAKO). For xenotransplantation of hiPSCs, $1 \times 10^6$ cells in 0.1 mL of cold Hank's balanced salt solution (HBSS) (Life Technologies) were injected into the right-side testis using a Hamilton syringe (Sigma-Aldrich) after exteriorization of the testes through a hypogastric incision. The mice were examined daily, and tumors were extracted at 10 weeks after surgery. Teratoma samples were resected and fixed with 4% paraformaldehyde; the tissues were paraffin embedded and stained with H&E according to standard procedures.

### Statistical Analysis

Statistical analyses were performed using Student's t test to compare two sample means. The analyses of multiple groups (i.e., more than two groups) were performed using one- and two-way ANOVAs with the XLSTAT software program (Addinsoft, Paris, France) and StatPlus software program (AnalystSoft, Walnut, CA, USA). Statistical significance was set at $^*p < 0.05$ or $^{**}p < 0.01$ for all tests. The data shown are representative examples of two independent experiments.

## SUPPLEMENTAL INFORMATION

Supplemental Information includes eight figures and can be found with this article online at https://doi.org/10.1016/j.ymthe.2018.06.005.

## AUTHOR CONTRIBUTIONS

Study Design, Y.N.; Study Conduct, Y.N.; Data Collection, Y.N.; Data Analysis, Y.N.; Data Interpretation, Y.N., C.M.-S., H.N., T.O.; Drafting of Manuscript, Y.N., T.O.; Revising Manuscript Content, Y.N., C.M.-S., H.N., T.O.; Approving Final Version of Manuscript, Y.N., C.M.-S., H.N., T.O.; and Y.N. takes responsibility for the integrity of all data analyses.

## CONFLICTS OF INTEREST

The authors declare no conflict of interest.

## ACKNOWLEDGMENTS

We thank the RIKEN Center for Developmental Biology, a group of the late Dr. Yoshiki Sasai, for help with methods that were indispensable to start this study. We thank Tomoko Shikata (Tokushima University), Masae Morio (Osaka University), Megumi Araki (Osaka University), and Naomi Kakazu (University of the Ryukyus) for the office processing, and Saki Uema, Yuka Onishi, Maki Higa, Youichi Toyokawa, Yuki Kawahira, and Saori Adaniya (University of the Ryukyus) for technical support. We thank Kazumi Sagayama (Center for Research Administration and Collaboration, Tokushima University); Kenji Okonogi, Toshihiko Matsuhashi, and Hideo Takasu (Office for University-Industry Collaboration, Osaka University); Yuki Tonooka (Organization for Research Promotion Research Planning Office, University of the Ryukyus); and Takeshi Arakawa and Youko Kobashigawa (Organization for the Promotion of Industry, University and Government Collaboration, University of the Ryukyus) for intellectual property consultation. We thank Masayoshi Tsukahara (Kyowa Hakko Kirin) for his expert technical advice concerning the cell culture methods. We thank Yasuyuki Kusuhara (Regience) for his expert technical advice. We also thank Dr. Mary Collins (Dean of Research, Okinawa Institute of Science and Technology [OIST] Graduate University), who gave us intellectual advice. This work was partially supported by the UIC (University-Industry Collaboration) Gap Fund at Osaka University; grants from the Adaptable and Seamless Technology Transfer Program through target-driven R&D of Japan (AS251Z00051N) under contract cooperative research with Kyowa Hakko Kirin Co., Ltd.; JSPS KAKENHI (16H07094); a joint fund of the Regional Collaboration Institute, University of the Ryukyus and The Bank of Okinawa, Ltd., under contract cooperative research with Regience; the Support Center for Advanced Medical Sciences, Institute of Health Biosciences, Division for Animal Research Resources and Genetic Engineering Support Center for Advanced Medical Sciences, Institute of Technology and Science, Tokushima University; and Research Laboratory Center, Faculty of Medicine and Institute for Animal Experiments, Faculty of Medicine, University of the Ryukyus.

## REFERENCES

1. Takahashi, K., Tanabe, K., Ohnuki, M., Narita, M., Ichisaka, T., Tomoda, K., and Yamanaka, S. (2007). Induction of pluripotent stem cells from adult human fibroblasts by defined factors. Cell *131*, 861–872.
2. Takahashi, K., Okita, K., Nakagawa, M., and Yamanaka, S. (2007). Induction of pluripotent stem cells from fibroblast cultures. Nat. Protoc. *2*, 3081–3089.
3. Ben-David, U., and Benvenisty, N. (2014). Chemical ablation of tumor-initiating human pluripotent stem cells. Nat. Protoc. *9*, 729–740.
4. Ben-David, U., and Benvenisty, N. (2011). The tumorigenicity of human embryonic and induced pluripotent stem cells. Nat. Rev. Cancer *11*, 268–277.
5. Ben-David, U., Kopper, O., and Benvenisty, N. (2012). Expanding the boundaries of embryonic stem cells. Cell Stem Cell *10*, 666–677.

6. Lee, A.S., Tang, C., Cao, F., Xie, X., van der Bogt, K., Hwang, A., Connolly, A.J., Robbins, R.C., and Wu, J.C. (2009). Effects of cell number on teratoma formation by human embryonic stem cells. Cell Cycle *8*, 2608–2612.

7. Hentze, H., Soong, P.L., Wang, S.T., Phillips, B.W., Putti, T.C., and Dunn, N.R. (2009). Teratoma formation by human embryonic stem cells: evaluation of essential parameters for future safety studies. Stem Cell Res. (Amst.) *2*, 198–210.

8. Wakao, S., Kitada, M., Kuroda, Y., Shigemoto, T., Matsuse, D., Akashi, H., Tanimura, Y., Tsuchiyama, K., Kikuchi, T., Goda, M., et al. (2011). Multilineage-differentiating stress-enduring (Muse) cells are a primary source of induced pluripotent stem cells in human fibroblasts. Proc. Natl. Acad. Sci. USA *108*, 9875–9880.

9. Noguchi, H., Saitoh, I., Tsugata, T., Kataoka, H., Watanabe, M., and Noguchi, Y. (2015). Induction of tissue-specific stem cells by reprogramming factors, and tissue-specific selection. Cell Death Differ. *22*, 145–155.

10. Ben-David, U., Gan, Q.F., Golan-Lev, T., Arora, P., Yanuka, O., Oren, Y.S., Leikin-Frenkel, A., Graf, M., Garippa, R., Boehringer, M., et al. (2013). Selective elimination of human pluripotent stem cells by an oleate synthesis inhibitor discovered in a high-throughput screen. Cell Stem Cell *12*, 167–179.

11. Shiraki, N., Shiraki, Y., Tsuyama, T., Obata, F., Miura, M., Nagae, G., Aburatani, H., Kume, K., Endo, F., and Kume, S. (2014). Methionine metabolism regulates maintenance and differentiation of human pluripotent stem cells. Cell Metab. *19*, 780–794.

12. Tohyama, S., Hattori, F., Sano, M., Hishiki, T., Nagahata, Y., Matsuura, T., Hashimoto, H., Suzuki, T., Yamashita, H., Satoh, Y., et al. (2013). Distinct metabolic flow enables large-scale purification of mouse and human pluripotent stem cell-derived cardiomyocytes. Cell Stem Cell *12*, 127–137.

13. Endo, A. (2010). A historical perspective on the discovery of statins. Proc. Jpn. Acad., Ser. B, Phys. Biol. Sci. *86*, 484–493.

14. Markovic-Plese, S., Singh, A.K., and Singh, I. (2008). Therapeutic potential of statins in multiple sclerosis: immune modulation, neuroprotection and neurorepair. Future Neurol. *3*, 153.

15. Kureishi, Y., Luo, Z., Shiojima, I., Bialik, A., Fulton, D., Lefer, D.J., Sessa, W.C., and Walsh, K. (2000). The HMG-CoA reductase inhibitor simvastatin activates the protein kinase Akt and promotes angiogenesis in normocholesterolemic animals. Nat. Med. *6*, 1004–1010.

16. Nakashima, Y., and Haneji, T. (2013). Stimulation of osteoclast formation by RANKL requires interferon regulatory factor-4 and is inhibited by simvastatin in a mouse model of bone loss. PLoS ONE *8*, e72033.

17. Mundy, G., Garrett, R., Harris, S., Chan, J., Chen, D., Rossini, G., Boyce, B., Zhao, M., and Gutierrez, G. (1999). Stimulation of bone formation in vitro and in rodents by statins. Science *286*, 1946–1949.

18. Goldstein, J.L., and Brown, M.S. (2015). A century of cholesterol and coronaries: from plaques to genes to statins. Cell *161*, 161–172.

19. Cannon, C.P., Braunwald, E., McCabe, C.H., Rader, D.J., Rouleau, J.L., Belder, R., Joyal, S.V., Hill, K.A., Pfeffer, M.A., and Skene, A.M.; Pravastatin or Atorvastatin Evaluation and Infection Therapy-Thrombolysis in Myocardial Infarction 22 Investigators (2004). Intensive versus moderate lipid lowering with statins after acute coronary syndromes. N. Engl. J. Med. *350*, 1495–1504.

20. Ridker, P.M., Cannon, C.P., Morrow, D., Rifai, N., Rose, L.M., McCabe, C.H., Pfeffer, M.A., and Braunwald, E.; Pravastatin or Atorvastatin Evaluation and Infection Therapy-Thrombolysis in Myocardial Infarction 22 (PROVE IT-TIMI 22) Investigators (2005). C-reactive protein levels and outcomes after statin therapy. N. Engl. J. Med. *352*, 20–28.

21. Lamon-Fava, S. (2013). Statins and lipid metabolism: an update. Curr. Opin. Lipidol. *24*, 221–226.

22. Kato, R., Matsumoto, M., Sasaki, H., Joto, R., Okada, M., Ikeda, Y., Kanie, K., Suga, M., Kinehara, M., Yanagihara, K., Liu, Y., et al. (2016). Parametric analysis of colony morphology of non-labelled live human pluripotent stem cells for cell quality control. Sci. Rep. *6*, 34009.

23. Kobayashi, M., Sugihara, N., Ise, H., Omasa, T., and Negishi, N. (2004). Real time monitoring of drug metabolic enzyme response inside human hepatoma GS-3A4-HepG2 cells by means of electrochemical impedance measurement. Polym. Adv. Technol. *15*, 232–243.

24. Ohgushi, M., Matsumura, M., Eiraku, M., Murakami, K., Aramaki, T., Nishiyama, A., Muguruma, K., Nakano, T., Suga, H., Ueno, M., et al. (2010). Molecular pathway and cell state responsible for dissociation-induced apoptosis in human pluripotent stem cells. Cell Stem Cell *7*, 225–239.

25. Watanabe, K., Ueno, M., Kamiya, D., Nishiyama, A., Matsumura, M., Wataya, T., Takahashi, J.B., Nishikawa, S., Nishikawa, S., Muguruma, K., and Sasai, Y. (2007). A ROCK inhibitor permits survival of dissociated human embryonic stem cells. Nat. Biotechnol. *25*, 681–686.

26. Wang, W., Foley, K., Shan, X., Wang, S., Eaton, S., Nagaraj, V.J., Wiktor, P., Patel, U., and Tao, N. (2011). Single cells and intracellular processes studied by a plasmonic-based electrochemical impedance microscopy. Nat. Chem. *3*, 249–255.

27. Xu, C., Inokuma, M.S., Denham, J., Golds, K., Kundu, P., Gold, J.D., and Carpenter, M.K. (2001). Feeder-free growth of undifferentiated human embryonic stem cells. Nat. Biotechnol. *19*, 971–974.

28. Nakashima, Y., Miyagi-Shiohira, C., Kobayashi, N., Saitoh, I., Watanabe, M., and Noguchi, H. (2017). A proteome analysis of pig pancreatic islets and exocrine tissue by liquid chromatography with tandem mass spectrometry. Islets *9*, 159–176.

29. Yamana, R., Iwasaki, M., Wakabayashi, M., Nakagawa, M., Yamanaka, S., and Ishihama, Y. (2013). Rapid and deep profiling of human induced pluripotent stem cell proteome by one-shot NanoLC-MS/MS analysis with meter-scale monolithic silica columns. J. Proteome Res. *12*, 214–221.

30. Sarkar, P., Randall, S.M., Muddiman, D.C., and Rao, B.M. (2012). Targeted proteomics of the secretory pathway reveals the secretome of mouse embryonic fibroblasts and human embryonic stem cells. Mol. Cell. Proteomics *11*, 1829–1839.

31. Gharbi, S.I., Zvelebil, M.J., Shuttleworth, S.J., Hancox, T., Saghir, N., Timms, J.F., and Waterfield, M.D. (2007). Exploring the specificity of the PI3K family inhibitor LY294002. Biochem. J. *404*, 15–21.

32. Watanabe, K., Ueno, M., Kamiya, D., Nishiyama, A., Matsumura, M., Wataya, T., Takahashi, J.B., Nishikawa, S., Nishikawa, S., Muguruma, K., and Sasai, Y. (2007). A ROCK inhibitor permits survival of dissociated human embryonic stem cells. Nat. Biotechnol. *25*, 681–686.

33. Ogata, Y., Takahashi, M., Takeuchi, K., Ueno, S., Mano, H., Ookawara, S., Kobayashi, E., Ikeda, U., and Shimada, K. (2002). Fluvastatin induces apoptosis in rat neonatal cardiac myocytes: a possible mechanism of statin-attenuated cardiac hypertrophy. J. Cardiovasc. Pharmacol. *40*, 907–915.

34. Song, X.J., Yang, C.Y., Liu, B., Wei, Q., Korkor, M.T., Liu, J.Y., and Yang, P. (2011). Atorvastatin inhibits myocardial cell apoptosis in a rat model with post-myocardial infarction heart failure by downregulating ER stress response. Int. J. Med. Sci. *8*, 564–572.

35. Leone, M., Magadum, A., and Engel, F.B. (2015). Cardiomyocyte proliferation in cardiac development and regeneration: a guide to methodologies and interpretations. Am. J. Physiol. Heart Circ. Physiol. *309*, H1237–H1250.

36. Dolezalova, D., Mraz, M., Barta, T., Plevova, K., Vinarsky, V., Holubcova, Z., Jaros, J., Dvorak, P., Pospisilova, S., and Hampl, A. (2012). MicroRNAs regulate p21(Waf1/Cip1) protein expression and the DNA damage response in human embryonic stem cells. Stem Cells *30*, 1362–1372.

37. Samuels, Y., Wang, Z., Bardelli, A., Silliman, N., Ptak, J., Szabo, S., Yan, H., Gazdar, A., Powell, S.M., Riggins, G.J., et al. (2004). High frequency of mutations of the PIK3CA gene in human cancers. Science *304*, 554.

38. Karakas, B., Bachman, K.E., and Park, B.H. (2006). Mutation of the PIK3CA oncogene in human cancers. Br. J. Cancer *94*, 455–459.

39. Hao, Y., Samuels, Y., Li, Q., Krokowski, D., Guan, B.J., Wang, C., Jin, Z., Dong, B., Cao, B., Feng, X., et al. (2016). Oncogenic PIK3CA mutations reprogram glutamine metabolism in colorectal cancer. Nat. Commun. *7*, 11971.

40. Janiszewska, M., Liu, L., Almendro, V., Kuang, Y., Paweletz, C., Sakr, R.A., Weigelt, B., Hanker, A.B., Chandarlapaty, S., King, T.A., et al. (2015). In situ single-cell analysis identifies heterogeneity for PIK3CA mutation and HER2 amplification in HER2-positive breast cancer. Nat. Genet. *47*, 1212–1219.

41. Van Keymeulen, A., Lee, M.Y., Ousset, M., Brohée, S., Rorive, S., Giraddi, R.R., Wuidart, A., Bouvencourt, G., Dubois, C., Salmon, I., et al. (2015). Reactivation of multipotency by oncogenic PIK3CA induces breast tumour heterogeneity. Nature *525*, 119–123.

42. Betteridge, D.J., and Carmena, R. (2016). The diabetogenic action of statins—mechanisms and clinical implications. Nat. Rev. Endocrinol. *12*, 99–110.

43. Carbia-Nagashima, A., Gerez, J., Perez-Castro, C., Paez-Pereda, M., Silberstein, S., Stalla, G.K., Holsboer, F., and Arzt, E. (2007). RSUME, a small RWD-containing protein, enhances SUMO conjugation and stabilizes HIF-1alpha during hypoxia. Cell *131*, 309–323.

44. Seeler, J.S., and Dejean, A. (2017). SUMO and the robustness of cancer. Nat. Rev. Cancer *17*, 184–197.

45. Fu, J., and Taubman, M.B. (2013). EGLN3 inhibition of NF-κB is mediated by prolyl hydroxylase-independent inhibition of IκB kinase γ ubiquitination. Mol. Cell. Biol. *33*, 3050–3061.

46. Fu, J., and Taubman, M.B. (2010). Prolyl hydroxylase EGLN3 regulates skeletal myoblast differentiation through an NF-kappaB-dependent pathway. J. Biol. Chem. *285*, 8927–8935.

47. Wang, X., Wang, G., Shi, Y., Sun, L., Gorczynski, R., Li, Y.J., Xu, Z., and Spaner, D.E. (2016). PPAR-delta promotes survival of breast cancer cells in harsh metabolic conditions. Oncogenesis *5*, e232.

48. Jacobsen, W., Kuhn, B., Soldner, A., Kirchner, G., Sewing, K.F., Kollman, P.A., Benet, L.Z., and Christians, U. (2000). Lactonization is the critical first step in the disposition of the 3-hydroxy-3-methylglutaryl-CoA reductase inhibitor atorvastatin. Drug Metab. Dispos. *28*, 1369–1378.

49. Singh, R.P., Gu, M., and Agarwal, R. (2008). Silibinin inhibits colorectal cancer growth by inhibiting tumor cell proliferation and angiogenesis. Cancer Res. *68*, 2043–2050.

50. García-Maceira, P., and Mateo, J. (2009). Silibinin inhibits hypoxia-inducible factor-1alpha and mTOR/p70S6K/4E-BP1 signalling pathway in human cervical and hepatoma cancer cells: implications for anticancer therapy. Oncogene *28*, 313–324.

51. Siegel, A.B., and Stebbing, J. (2013). Milk thistle: early seeds of potential. Lancet Oncol *14*, 929–930.

52. Li, D., Hu, J., Wang, T., Zhang, X., Liu, L., Wang, H., Wu, Y., Xu, D., and Wen, F. (2016). Silymarin attenuates cigarette smoke extract-induced inflammation via simultaneous inhibition of autophagy and ERK/p38 MAPK pathway in human bronchial epithelial cells. Sci Rep *6*, 37751.

53. Fu, B., Xue, J., Li, Z., Shi, X., Jiang, B.H., and Fang, J. (2007). Chrysin inhibits expression of hypoxia-inducible factor-1alpha through reducing hypoxia-inducible factor-1alpha stability and inhibiting its protein synthesis. Mol. Cancer Ther. *6*, 220–226.

54. Wu, J., Ocampo, A., and Belmonte, J.C.I. (2016). Cellular metabolism and induced pluripotency. Cell *166*, 1371–1385.

55. Mathieu, J., Zhou, W., Xing, Y., Sperber, H., Ferreccio, A., Agoston, Z., Kuppusamy, K.T., Moon, R.T., and Ruohola-Baker, H. (2014). Hypoxia-inducible factors have distinct and stage-specific roles during reprogramming of human cells to pluripotency. Cell Stem Cell *14*, 592–605.

56. Ahmadian, M., Suh, J.M., Hah, N., Liddle, C., Atkins, A.R., Downes, M., and Evans, R.M. (2013). PPARγ signaling and metabolism: the good, the bad and the future. Nat. Med. *19*, 557–566.

57. de Lera, A.R., Bourguet, W., Altucci, L., and Gronemeyer, H. (2007). Design of selective nuclear receptor modulators: RAR and RXR as a case study. Nat. Rev. Drug Discov. *6*, 811–820.

58. Roberts-Thomson, S.J. (2000). Peroxisome proliferator-activated receptors in tumorigenesis: targets of tumour promotion and treatment. Immunol. Cell Biol. *78*, 436–441.

59. Cunningham, T.J., and Duester, G. (2015). Mechanisms of retinoic acid signalling and its roles in organ and limb development. Nat. Rev. Mol. Cell Biol. *16*, 110–123.

60. Michalik, L., Desvergne, B., and Wahli, W. (2004). Peroxisome-proliferator-activated receptors and cancers: complex stories. Nat. Rev. Cancer *4*, 61–70.

61. Daynes, R.A., and Jones, D.C. (2002). Emerging roles of PPARs in inflammation and immunity. Nat. Rev. Immunol. *2*, 748–759.

62. Chou, F.S., Wang, P.S., Kulp, S., and Pinzone, J.J. (2007). Effects of thiazolidinediones on differentiation, proliferation, and apoptosis. Mol. Cancer Res. *5*, 523–530.

63. Peters, J.M., Shah, Y.M., and Gonzalez, F.J. (2012). The role of peroxisome proliferator-activated receptors in carcinogenesis and chemoprevention. Nat. Rev. Cancer *12*, 181–195.

64. Ben-David, U., Nudel, N., and Benvenisty, N. (2013). Immunologic and chemical targeting of the tight-junction protein Claudin-6 eliminates tumorigenic human pluripotent stem cells. Nat. Commun. *4*, 1992.

65. Fong, C.Y., Peh, G.S., Gauthaman, K., and Bongso, A. (2009). Separation of SSEA-4 and TRA-1-60 labelled undifferentiated human embryonic stem cells from a heterogeneous cell population using magnetic-activated cell sorting (MACS) and fluorescence-activated cell sorting (FACS). Stem Cell Rev. *5*, 72–80.

66. Tang, C., Lee, A.S., Volkmer, J.P., Sahoo, D., Nag, D., Mosley, A.R., Inlay, M.A., Ardehali, R., Chavez, S.L., Pera, R.R., et al. (2011). An antibody against SSEA-5 glycan on human pluripotent stem cells enables removal of teratoma-forming cells. Nat. Biotechnol. *29*, 829–834.

67. Wang, Y.C., Nakagawa, M., Garitaonandia, I., Slavin, I., Altun, G., Lacharite, R.M., Nazor, K.L., Tran, H.T., Lynch, C.L., Leonardo, T.R., et al. (2011). Specific lectin biomarkers for isolation of human pluripotent stem cells identified through array-based glycomic analysis. Cell Res. *21*, 1551–1563.

68. Choo, A.B., Tan, H.L., Ang, S.N., Fong, W.J., Chin, A., Lo, J., Zheng, L., Hentze, H., Philp, R.J., Oh, S.K., and Yap, M. (2008). Selection against undifferentiated human embryonic stem cells by a cytotoxic antibody recognizing podocalyxin-like protein-1. Stem Cells *26*, 1454–1463.

69. Tan, H.L., Fong, W.J., Lee, E.H., Yap, M., and Choo, A. (2009). mAb 84, a cytotoxic antibody that kills undifferentiated human embryonic stem cells via oncosis. Stem Cells *27*, 1792–1801.

70. Blum, B., Bar-Nur, O., Golan-Lev, T., and Benvenisty, N. (2009). The anti-apoptotic gene survivin contributes to teratoma formation by human embryonic stem cells. Nat. Biotechnol. *27*, 281–287.

71. Menendez, S., Camus, S., Herreria, A., Paramonov, I., Morera, L.B., Collado, M., Pekarik, V., Maceda, I., Edel, M., Consiglio, A., et al. (2012). Increased dosage of tumor suppressors limits the tumorigenicity of iPS cells without affecting their pluripotency. Aging Cell *11*, 41–50.

72. Rong, Z., Fu, X., Wang, M., and Xu, Y. (2012). A scalable approach to prevent teratoma formation of human embryonic stem cells. J. Biol. Chem. *287*, 32338–32345.

73. Schuldiner, M., Itskovitz-Eldor, J., and Benvenisty, N. (2003). Selective ablation of human embryonic stem cells expressing a "suicide" gene. Stem Cells *21*, 257–265.

74. Eiges, R., Schuldiner, M., Drukker, M., Yanuka, O., Itskovitz-Eldor, J., and Benvenisty, N. (2001). Establishment of human embryonic stem cell-transfected clones carrying a marker for undifferentiated cells. Curr. Biol. *11*, 514–518.

75. Chung, S., Shin, B.S., Hedlund, E., Pruszak, J., Ferree, A., Kang, U.J., Isacson, O., and Kim, K.S. (2006). Genetic selection of sox1GFP-expressing neural precursors removes residual tumorigenic pluripotent stem cells and attenuates tumor formation after transplantation. J. Neurochem. *97*, 1467–1480.

76. Huber, I., Itzhaki, I., Caspi, O., Arbel, G., Tzukerman, M., Gepstein, A., Habib, M., Yankelson, L., Kehat, I., and Gepstein, L. (2007). Identification and selection of cardiomyocytes during human embryonic stem cell differentiation. FASEB J. *21*, 2551–2563.

77. Kuang, Y., Miki, K., Parr, C.J.C., Hayashi, K., Takei, I., Li, J., Iwasaki, M., Nakagawa, M., Yoshida, Y., and Saito, H. (2017). Efficient, selective removal of human pluripotent stem cells via ecto-alkaline phosphatase-mediated aggregation of synthetic peptides. Cell Chem. Biol. *24*, 685–694.e4.

78. Masuda, S., Miyagawa, S., Sougawa, N., and Sawa, Y. (2015). CD30-targeting immunoconjugates and bystander effects. Nat. Rev. Clin. Oncol. *12*, 245.

79. Dabir, D.V., Hasson, S.A., Setoguchi, K., Johnson, M.E., Wongkongkathep, P., Douglas, C.J., Zimmerman, J., Damoiseaux, R., Teitell, M.A., and Koehler, C.M. (2013). A small molecule inhibitor of redox-regulated protein translocation into mitochondria. Dev. Cell *25*, 81–92.

80. Lee, M.O., Moon, S.H., Jeong, H.C., Yi, J.Y., Lee, T.H., Shim, S.H., Rhee, Y.H., Lee, S.H., Oh, S.J., Lee, M.Y., et al. (2013). Inhibition of pluripotent stem cell-derived teratoma formation by small molecules. Proc. Natl. Acad. Sci. USA *110*, E3281–E3290.

81. Germain, N.D., Hartman, N.W., Cai, C., Becker, S., Naegele, J.R., and Grabel, L.B. (2012). Teratocarcinoma formation in embryonic stem cell-derived neural progenitor hippocampal transplants. Cell Transplant. *21*, 1603–1611.

82. Roy, N.S., Cleren, C., Singh, S.K., Yang, L., Beal, M.F., and Goldman, S.A. (2006). Functional engraftment of human ES cell-derived dopaminergic neurons enriched by coculture with telomerase-immortalized midbrain astrocytes. Nat. Med. *12*, 1259–1268.

83. Wernig, M., Benninger, F., Schmandt, T., Rade, M., Tucker, K.L., Büssow, H., Beck, H., and Brüstle, O. (2004). Functional integration of embryonic stem cell-derived neurons in vivo. J. Neurosci. *24*, 5258–5268.

84. Xie, X., Cao, F., Sheikh, A.Y., Li, Z., Connolly, A.J., Pei, X., Li, R.K., Robbins, R.C., and Wu, J.C. (2007). Genetic modification of embryonic stem cells with VEGF enhances cell survival and improves cardiac function. Cloning Stem Cells *9*, 549–563.

85. Kato, R., Matsumoto, M., Sasaki, H., Joto, R., Okada, M., Ikeda, Y., Kanie, K., Suga, M., Kinehara, M., Yanagihara, K., et al. (2016). Parametric analysis of colony morphology of non-labelled live human pluripotent stem cells for cell quality control. Sci. Rep. *6*, 34009.

86. Kim, M.H., Masuda, E., and Kino-oka, M. (2014). Kinetic analysis of deviation from the undifferentiated state in colonies of human induced pluripotent stem cells on feeder layers. Biotechnol. Bioeng. *111*, 1128–1138.

87. Hirota, T., and Ieiri, I. (2015). Drug-drug interactions that interfere with statin metabolism. Expert Opin. Drug Metab. Toxicol. *11*, 1435–1447.

88. Corsini, A., Bellosta, S., Baetta, R., Fumagalli, R., Paoletti, R., and Bernini, F. (1999). New insights into the pharmacodynamic and pharmacokinetic properties of statins. Pharmacol. Ther. *84*, 413–428.

89. Yuan, T.L., and Cantley, L.C. (2008). PI3K pathway alterations in cancer: variations on a theme. Oncogene *27*, 5497–5510.

90. Brandes, R.P. (2005). Statin-mediated inhibition of Rho: only to get more NO? Circ. Res. *96*, 927–929.

91. Khwaja, A., O'Connolly, J., and Hendry, B.M. (2000). Prenylation inhibitors in renal disease. Lancet *355*, 741–744.

92. Schmidmaier, R., Baumann, P., Simsek, M., Dayyani, F., Emmerich, B., and Meinhardt, G. (2004). The HMG-CoA reductase inhibitor simvastatin overcomes cell adhesion-mediated drug resistance in multiple myeloma by geranylgeranylation of Rho protein and activation of Rho kinase. Blood *104*, 1825–1832.

93. Luo, H.R., Hattori, H., Hossain, M.A., Hester, L., Huang, Y., Lee-Kwon, W., Donowitz, M., Nagata, E., and Snyder, S.H. (2003). Akt as a mediator of cell death. Proc. Natl. Acad. Sci. USA *100*, 11712–11717.

94. Bellacosa, A., Testa, J.R., Moore, R., and Larue, L. (2004). A portrait of AKT kinases: human cancer and animal models depict a family with strong individualities. Cancer Biol. Ther. *3*, 268–275.

95. Barnett, S.F., Bilodeau, M.T., and Lindsley, C.W. (2005). The Akt/PKB family of protein kinases: a review of small molecule inhibitors and progress towards target validation. Curr. Top. Med. Chem. *5*, 109–125.

96. West, K.A., Castillo, S.S., and Dennis, P.A. (2002). Activation of the PI3K/Akt pathway and chemotherapeutic resistance. Drug Resist. Updat *5*, 234–248.

97. Schieber, M., and Chandel, N.S. (2014). ROS function in redox signaling and oxidative stress. Curr. Biol. *24*, R453–R462.

98. Prigione, A., Fauler, B., Lurz, R., Lehrach, H., and Adjaye, J. (2010). The senescence-related mitochondrial/oxidative stress pathway is repressed in human induced pluripotent stem cells. Stem Cells *28*, 721–733.

99. Folmes, C.D., Nelson, T.J., Martinez-Fernandez, A., Arrell, D.K., Lindor, J.Z., Dzeja, P.P., Ikeda, Y., Perez-Terzic, C., and Terzic, A. (2011). Somatic oxidative bioenergetics transitions into pluripotency-dependent glycolysis to facilitate nuclear reprogramming. Cell Metab. *14*, 264–271.

100. Oh, S.K., Kim, H.S., Ahn, H.J., Seol, H.W., Kim, Y.Y., Park, Y.B., Yoon, C.J., Kim, D.W., Kim, S.H., and Moon, S.Y. (2005). Derivation and characterization of new human embryonic stem cell lines: SNUhES1, SNUhES2, and SNUhES3. Stem Cells *23*, 211–219.

101. St John, J.C., Ramalho-Santos, J., Gray, H.L., Petrosko, P., Rawe, V.Y., Navara, C.S., Simerly, C.R., and Schatten, G.P. (2005). The expression of mitochondrial DNA transcription factors during early cardiomyocyte in vitro differentiation from human embryonic stem cells. Cloning Stem Cells *7*, 141–153.

102. Cho, Y.M., Kwon, S., Pak, Y.K., Seol, H.W., Choi, Y.M., Park, D.J., Park, K.S., and Lee, H.K. (2006). Dynamic changes in mitochondrial biogenesis and antioxidant enzymes during the spontaneous differentiation of human embryonic stem cells. Biochem. Biophys. Res. Commun. *348*, 1472–1478.

103. Facucho-Oliveira, J.M., Alderson, J., Spikings, E.C., Egginton, S., and St John, J.C. (2007). Mitochondrial DNA replication during differentiation of murine embryonic stem cells. J. Cell Sci. *120*, 4025–4034.

104. Mathieu, J., Zhang, Z., Zhou, W., Wang, A.J., Heddleston, J.M., Pinna, C.M., Hubaud, A., Stadler, B., Choi, M., Bar, M., et al. (2011). HIF induces human embryonic stem cell markers in cancer cells. Cancer Res. *71*, 4640–4652.

105. Saito, K., Kondo, E., and Matsushita, M. (2011). MicroRNA 130 family regulates the hypoxia response signal through the P-body protein DDX6. Nucleic Acids Res. *39*, 6086–6099.

106. Semenza, G.L. (2003). Targeting HIF-1 for cancer therapy. Nat. Rev. Cancer *3*, 721–732.

107. Krishnan, J., Suter, M., Windak, R., Krebs, T., Felley, A., Montessuit, C., Tokarska-Schlattner, M., Aasum, E., Bogdanova, A., Perriard, E., et al. (2009). Activation of a HIF1alpha-PPARgamma axis underlies the integration of glycolytic and lipid anabolic pathways in pathologic cardiac hypertrophy. Cell Metab. *9*, 512–524.

108. Zhao, Y.Z., Liu, X.L., Shen, G.M., Ma, Y.N., Zhang, F.L., Chen, M.T., Zhao, H.L., Yu, J., and Zhang, J.W. (2014). Hypoxia induces peroxisome proliferator-activated receptor $\gamma$ expression via HIF-1-dependent mechanisms in HepG2 cell line. Arch. Biochem. Biophys. *543*, 40–47.

109. Blum, J.I., Bijli, K.M., Murphy, T.C., Kleinhenz, J.M., and Hart, C.M. (2016). Time-dependent PPAR$\gamma$ modulation of HIF-1$\alpha$ signaling in hypoxic pulmonary artery smooth muscle cells. Am. J. Med. Sci. *352*, 71–79.

110. Wang, Y., Lu, W., Yang, K., Wang, Y., Zhang, J., Jia, J., Yun, X., Tian, L., Chen, Y., Jiang, Q., et al. (2015). Peroxisome proliferator-activated receptor $\gamma$ inhibits pulmonary hypertension targeting store-operated calcium entry. J. Mol. Med. (Berl.) *93*, 327–342.

111. Hydbring, P., Malumbres, M., and Sicinski, P. (2016). Non-canonical functions of cell cycle cyclins and cyclin-dependent kinases. Nat. Rev. Mol. Cell Biol. *17*, 280–292.

112. Nakashima, Y., and Omasa, T. (2016). What kind of signaling maintains pluripotency and viability in human-induced pluripotent stem cells cultured on Laminin-511 with serum-free medium? Biores. Open Access *5*, 84–93.

113. Fruman, D.A., and Rommel, C. (2014). PI3K and cancer: lessons, challenges and opportunities. Nat. Rev. Drug Discov. *13*, 140–156.

114. Jiao, M., and Nan, K.J. (2012). Activation of PI3 kinase/Akt/HIF-1$\alpha$ pathway contributes to hypoxia-induced epithelial-mesenchymal transition and chemoresistance in hepatocellular carcinoma. Int. J. Oncol. *40*, 461–468.

115. Agani, F., and Jiang, B.H. (2013). Oxygen-independent regulation of HIF-1: novel involvement of PI3K/AKT/mTOR pathway in cancer. Curr. Cancer Drug Targets *13*, 245–251.

116. Chatterjee, A., Mondal, P., Ghosh, S., Mehta, V.S., and Sen, E. (2015). PPAR$\gamma$ regulated CIDEA affects pro-apoptotic responses in glioblastoma. Cell Death Dis. *1*, 15038.

117. Regazzetti, C., Peraldi, P., Grémeaux, T., Najem-Lendom, R., Ben-Sahra, I., Cormont, M., Bost, F., Le Marchand-Brustel, Y., Tanti, J.F., and Giorgetti-Peraldi, S. (2009). Hypoxia decreases insulin signaling pathways in adipocytes. Diabetes *58*, 95–103.

118. Sone, M., Morone, N., Nakamura, T., Tanaka, A., Okita, K., Woltjen, K., Nakagawa, M., Heuser, J.E., Yamada, Y., Yamanaka, S., and Yamamoto, T. (2017). Hybrid cellular metabolism coordinated by Zic3 and Esrrb synergistically enhances induction of naive pluripotency. Cell Metab *25*, 1103–1117.e6.

119. Burridge, P.W., Matsa, E., Shukla, P., Lin, Z.C., Churko, J.M., Ebert, A.D., Lan, F., Diecke, S., Huber, B., Mordwinkin, N.M., et al. (2014). Chemically defined generation of human cardiomyocytes. Nat. Methods *11*, 855–860.

120. Burridge, P.W., Keller, G., Gold, J.D., and Wu, J.C. (2012). Production of de novo cardiomyocytes: human pluripotent stem cell differentiation and direct reprogramming. Cell Stem Cell *10*, 16–28.

121. Kaluzhny, Y., Kandárová, H., Hayden, P., Kubilus, J., d'Argembeau-Thornton, L., and Klausner, M. (2011). Development of the EpiOcular(TM) eye irritation test for hazard identification and labelling of eye irritating chemicals in response to the requirements of the EU cosmetics directive and REACH legislation. Altern. Lab. Anim. *39*, 339–364.

122. Kaluzhny, Y., Kandárová, H., Handa, Y., DeLuca, J., Truong, T., Hunter, A., Kearney, P., d'Argembeau-Thornton, L., and Klausner, M. (2015). The EpiOcular Eye Irritation Test (EIT) for hazard identification and labelling of eye irritating chemicals: protocol optimisation for solid materials and the results after extended shipment. Altern. Lab. Anim. *43*, 101–127.

123. Hamasaki, M., Hashizume, Y., Yamada, Y., Katayama, T., Hohjoh, H., Fusaki, N., Nakashima, Y., Furuya, H., Haga, N., Takami, Y., and Era, T. (2012). Pathogenic mutation of ALK2 inhibits induced pluripotent stem cell reprogramming and maintenance: mechanisms of reprogramming and strategy for drug identification. Stem Cells *30*, 2437–2449.

124. Okita, K., Matsumura, Y., Sato, Y., Okada, A., Morizane, A., Okamoto, S., Hong, H., Nakagawa, M., Tanabe, K., Tezuka, K., et al. (2011). A more efficient method to generate integration-free human iPS cells. Nat. Methods *8*, 409–412.

YMTHE, Volume *26*

# Supplemental Information

# Atorvastatin Inhibits the HIF1α-PPAR Axis, Which Is Essential for Maintaining the Function of Human Induced Pluripotent Stem Cells

**Yoshiki Nakashima, Chika Miyagi-Shiohira, Hirofumi Noguchi, and Takeshi Omasa**

Figure S1

a

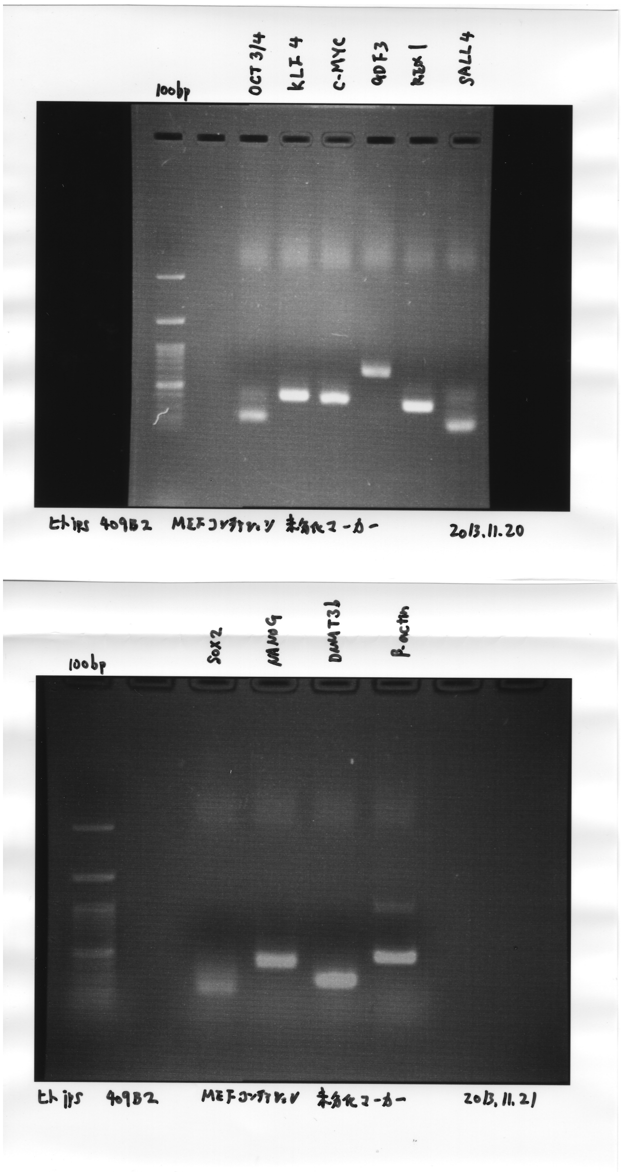


b

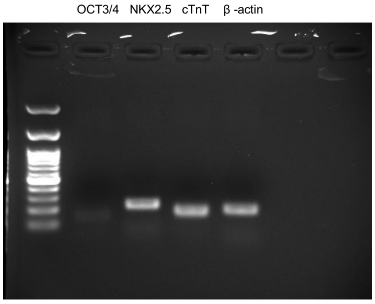


**Figure S1. Full unedited gel. (a)** Full-length images of the blots used for Figure 1d and (b) Figure S5c.

Figure S2

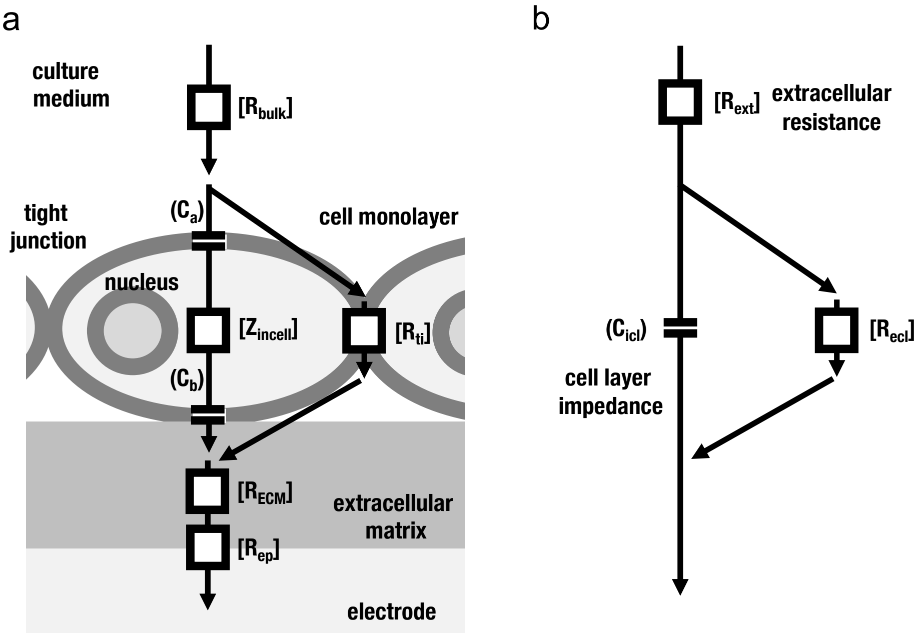


**Electric resistance value in direct current (d.c) circuit [$R_{DC}$],**
**Voltage [V], intensity of electric current [I]**
**$V=R_{DC}I$ [Ohm's law]**

**Electric resistance value in alternating current (a.c) circuit [$R_{AC}$],**
**a.c voltage [ΔV],**
**a.c intensity of electric current [ΔI]**
**$\Delta V=R_{AC}\Delta I$**
**<u>$R_{AC}=\Delta V/\Delta I$= Impedance values [Z]</u>**

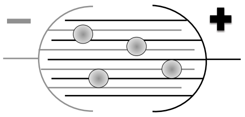

Cell index = $Z_{cell} - Z_0$

**(a) : Factors affecting Z**
**Resistance of the medium [$R_{bulk}$], Resistance of the tight junction [$R_{tj}$], Capacitance of the apical cell membrane ($C_a$), Capacitance of the basal cell membrane ($C_b$), impedance of intracellular element ($Z_{incell}$), Resistance of extracellular matrix [$R_{ECM}$], Resistance of electrode plate ($R_{ep}$)**
**(b) : Factor model affecting Z**
**Resistance of the extracellular element [$R_{ext}$], Resistance of the intercellular element [$R_{ecl}$], Capacitance of the cell monolayer ($C_{icl}$)**

**Figure S2. A circuit diagram for calculating the impedance values of hiPSCs and differentiated cells derived from hiPSCs.** (a) A configuration diagram of energizing circuit penetrating single cell layer of hiPSC under adhesion culture conditions. (b) A model diagram showing the method for calculating the impedance value of hiPSC under adhesive culture conditions.

Figure S3

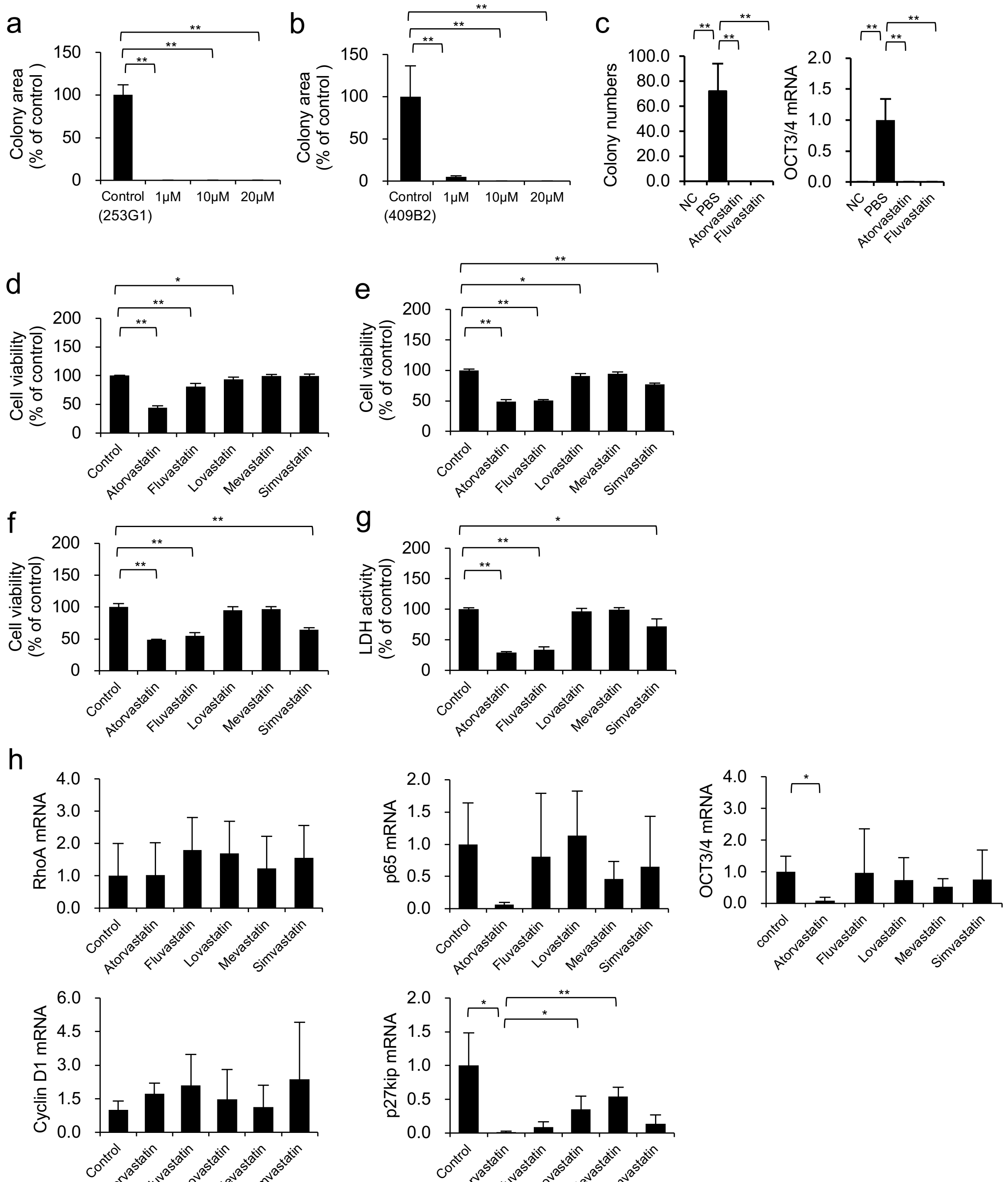


**Figure S3. Cell assays and mRNA expression analysis**. (a, b) Growth and viability assays of hiPSC lines 253G1 (a) and 409B2 (b) after 48 h of culture in the presence of 1, 10, 20 μM Atorvastatin. The cells were stained for AP. Colony areas per well were measured. The relative values are indicated. n = 3. Data represent mean ± S.D. *P < 0.05, **P < 0.01. (c) Left panel, the number of hiPSC colonies that were subjected to AP staining 4 days after the re-seeding of hiPSCs cultured under the same conditions as the teratoma experiments (Fig 2g) on MEF feeder—not mouse testis—and colony number/well were analyzed. n = 6. Data represent mean ± S.D. **P < 0.01. Right panel, a quantitative real-time PCR was performed to detect OCT3/4 mRNA (4 days after the re-seeding of hiPSCs cultured under the same conditions as the teratoma experiments [Fig 2g]) on MEF feeder—not mouse testis—and OCT3/4 mRNA expression level/well was analyzed. n = 6. Data represent the mean ± S.D. **P < 0.01. (d) Cell proliferation assays using Cell Counting Kit. (e) Cell count Reagent SF assay. (f) Assay of Neutral Red uptake. hiPSCs were analyzed after 48 h of culture in the presence of 20 μM Atorvastatin, Fluvastatin, Lovastatin, Mevastatin or Simvastatin. Each measurement was obtained using a microplate reader. (n = 3). Data represent mean ± S.D. *P < 0.05, **P < 0.01. (g) LDH assay of cytotoxicity in hiPSCs after 48 h of culture in the presence of 20 μM Atorvastatin, Fluvastatin, Lovastatin, Mevastatin or Simvastatin. Each measurement was obtained using a microplate reader. n = 3. Data represent mean ± S.D. *P < 0.05, **P < 0.01. (h) A quantitative real-time PCR analysis of RhoA, Cyclin D1, p65, p27kip and OCT3/4 mRNA in hiPSCs (201B7) after 24 h of culture in the presence of 10 μM Atorvastatin, Fluvastatin, Lovastatin, Mevastatin or Simvastatin. Data represent mean ± S.D. *P < 0.05.

Figure S4

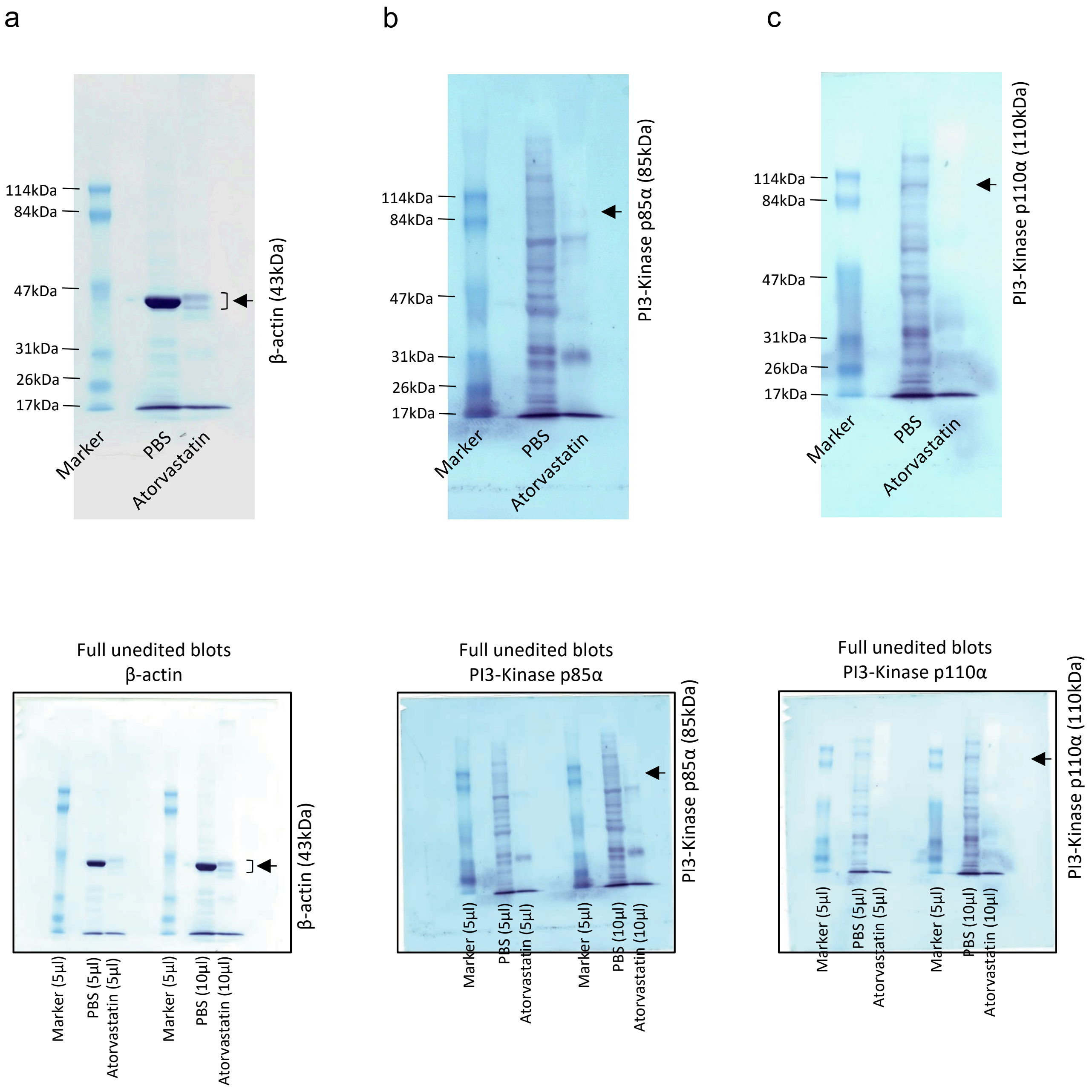


**Figure S4. Western blot assays of hiPSCs (201B7) after 24 h of culture in the absence or presence of 20 μM Atorvastatin.** (a) Top panel, full-length image of β-actin protein band photo sensitively detected on membrane by coloring. Bottom panel, Full unedited blots: Marker and β-actin. The same pair of samples flowed to the left (5 μl) and right (10 μl) of the membrane. (b) Top panel, full-length image of PI3-Kinase p85α protein band photo sensitively detected on membrane by coloring. PI3-Kinase p85α. Bottom panel, Full unedited blots: Marker and PI3-Kinase p85α. The same pair of samples flowed to the left (5 μl) and right (10 μl) of the membrane. (c) Top panel, full-length image of PI3-Kinase p110α protein band photo sensitively detected on membrane by coloring. PI3-Kinase p110α. Bottom panel, Full unedited blots: Marker and PI3-Kinase p110α. The same pair of samples flowed to the left (5 μl) and right (10 μl) of the membrane.

Figure S5

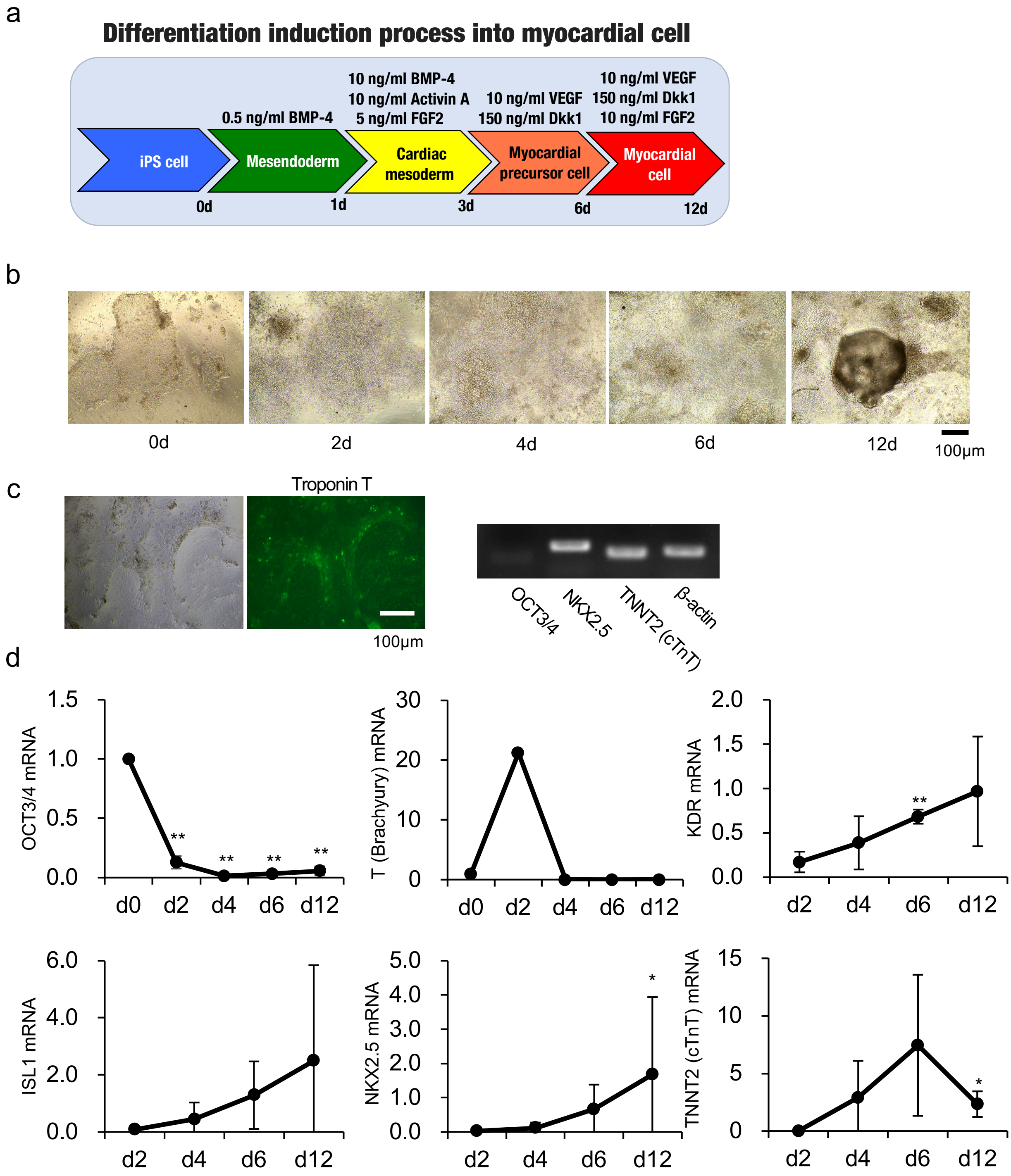


**Figure S5. The method for inducing the differentiation of myocardial cells from hiPSCs (201B7), and a cellular characteristic of the differentiation stage.** (a) A general myocardial cell differentiation induction protocol is shown. Representative medium addition factors and incubation period are shown. Differentiated cells at each stage are shown. (b) Microphotographs of 0, 2, 4, 6 and 12 d are shown after the initiation of differentiation induction. Scale bar = 100 μm. (c) Immunohistochemical staining method for Troponin T in myocardial cells differentiated from hiPSCs. Optical microscope images are shown (left is an optical microscope image, right is a fluorescence microscope image). Scale bar = 100 μm. Light panel, RT-PCR assay of OCT3/4, NKX2.5 and TNNT2 (cTnT) in myocardial cells differentiated from hiPSCs. (d) The mRNA expression levels of OCT3/4, T (Brachyury), KDR, ISL1, NKX2.5, TNNT2 (cTnT) at 0, 2, 4, 6 and 12 d after the initiation of differentiation induction are shown. The expression was calculated using the ΔΔCt method. The expression of the target gene was corrected by the expression of the housekeeping gene. The relative values are indicated. n = 3. Data represent the mean ± S.D. *P < 0.05, **P < 0.01.

Figure S6

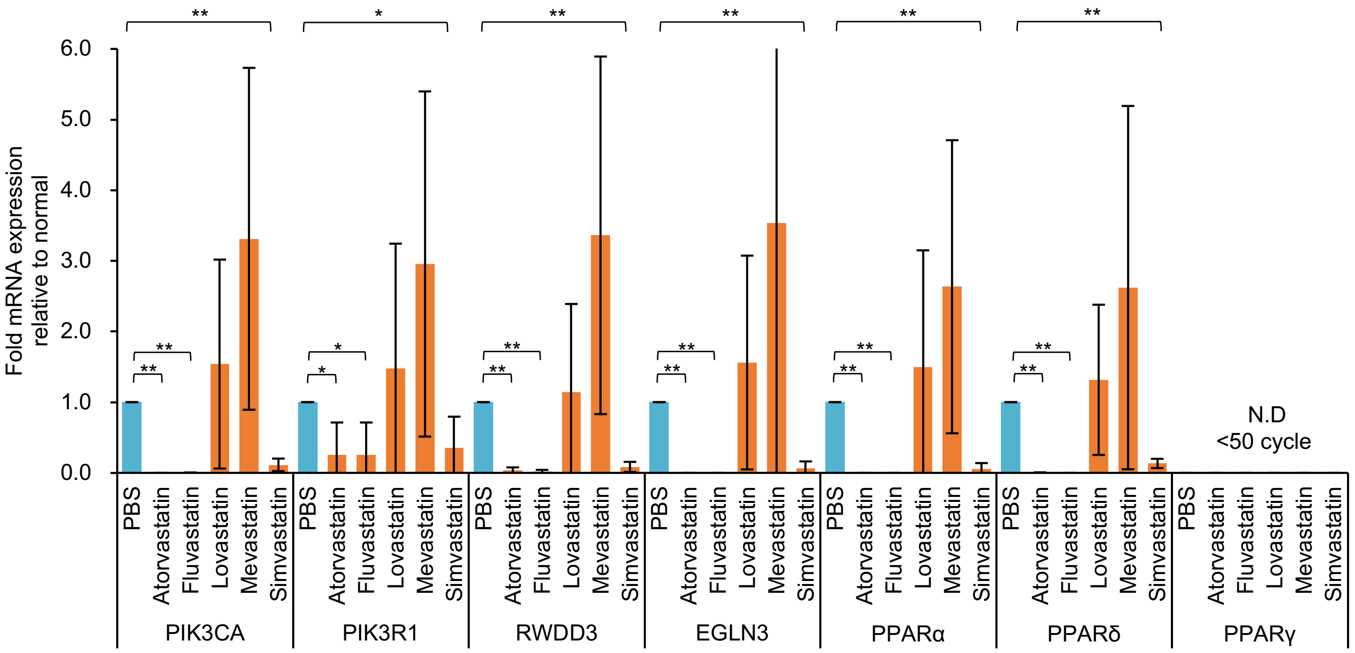


**Figure S6. The PI3K/AKT, HIF1α, NF-κB and PPAR signaling are involved in the effect of Atorvastatin on hiPSCs.** cDNA was synthesized using hiPSCs (201B7) administered PBS for 24 h and hiPSCs (201B7) administered 20 µM Atorvastatin, Fluvastatin, Lovastatin, Mevastatin and Simvastatin for 24 h. The mRNA expression level was calculated using the ΔΔCt method. The expression of the target gene was corrected by the expression of the housekeeping gene. First and second panels from the left, a quantitative real-time PCR analysis of the protein complex of PI3K. n = 4. Data represent mean ± S.D. **P < 0.01. Third and fourth panels from the left, a quantitative real-time PCR analysis of HIF1α and NF-κB signaling. n = 4. Data represent the mean ± S.D. **P < 0.01. 1st, 2nd and 3nd panels from the right, a quantitative real-time PCR analysis of PPARs signaling. n = 4. Data represent the mean ± S.D. **P < 0.01.

Figure S7

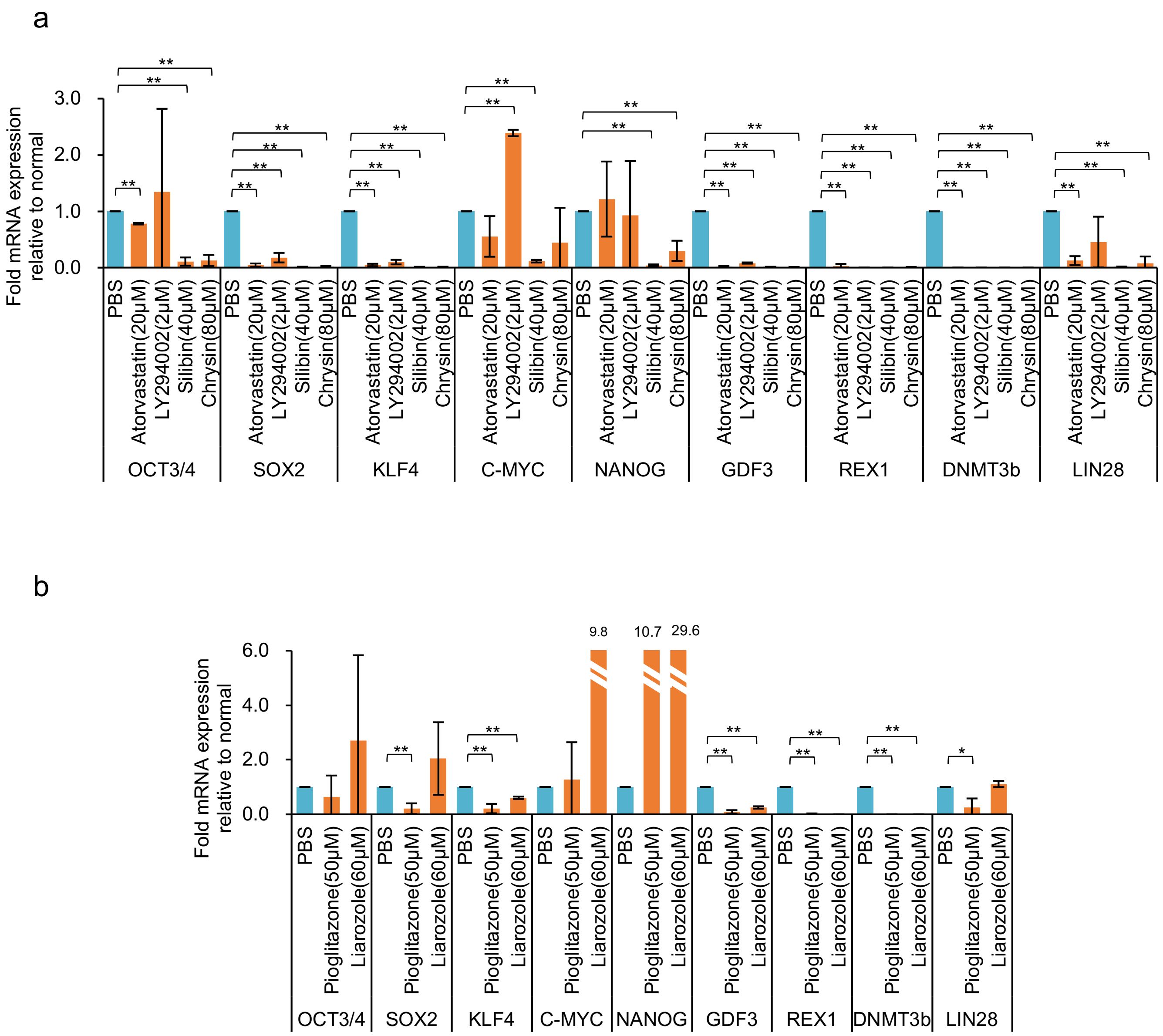


**Figure S7. Effect of various drugs on the expression of undifferentiated marker mRNA of hiPSC.** (a) The residual state of undifferentiated iPSCs are involved in the effect of Atorvastatin, LY294002, Silibin and Chrysin on hiPSCs. cDNA was synthesized using hiPSCs (201B7) administered PBS for 6 h and hiPSCs (201B7) administered 20 μM Atorvastatin, 2μM LY294002, 40 μM Silibin and 80 μM Chrysin for 6 h. The expression was calculated using the ΔΔCt method. The expression of the target gene was corrected by the expression of the housekeeping gene. A quantitative real-time PCR analysis of undifferentiated marker. n = 3. Data represent the mean ± S.D. **P < 0.01. (b) The residual state of undifferentiated iPSCs are involved in the effect of Pioglitazone and Liarozole on hiPSCs. cDNA was synthesized using hiPSCs (201B7) administered PBS for 6 h and hiPSCs (201B7) administered 50 μM Pioglitazone and 60 μM Liarozole for 6 h. The expression was calculated using the ΔΔCt method. The expression of the target gene was corrected by the expression of the housekeeping gene. A quantitative real-time PCR analysis of undifferentiated markers. n = 3. Data represent the mean ± S.D. *P < 0.05, **P < 0.01.

Figure S8

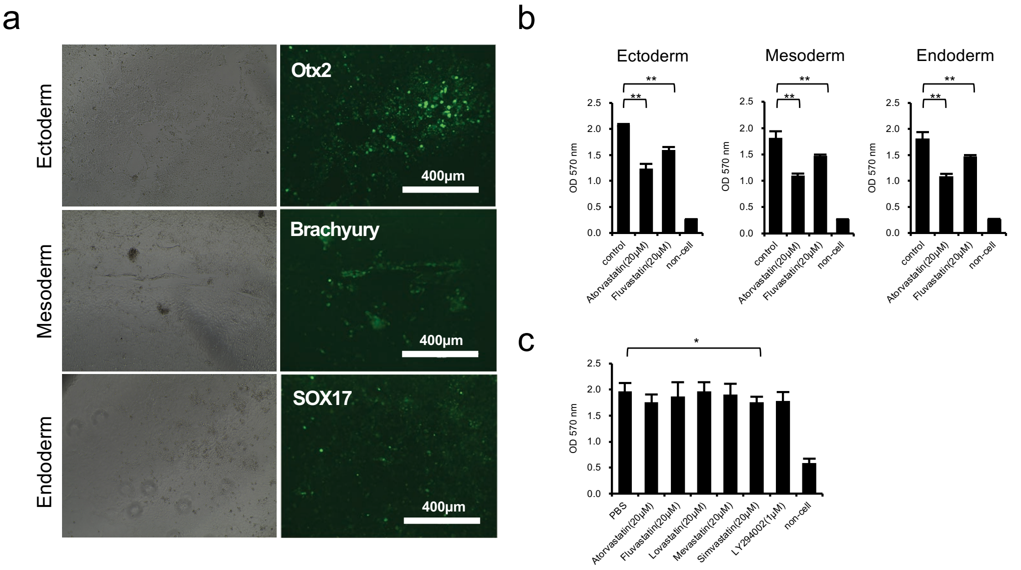


**Figure S8. The effect of statins on the viable cell activity of endoderm, mesoderm, ectoderm and myocardial cells.** (a) Top panel, Immunohistochemical staining of Otx2 on ectoderm differentiated from hiPSCs. Middle panel, Immunohistochemical staining of Brachyury in mesoderm differentiated from hiPSCs. Lower panel, Immunohistochemical staining of SOX17 in endoderm differentiated from hiPSCs. Optical microscope images are shown (left is an optical microscope image, right is a fluorescence microscope image). Scale bar = 400 μm. (b) Left panel, cell viability assays (MTT assay) of ectoderm differentiated from hiPSCs (201B7) after 48 h of culture in the presence of 20 μM Atorvastatin and 20 μM Fluvastatin. Each measurement was performed using a microplate reader. The relative values are indicated. n = 4. Data represent the mean ± S.D. **P < 0.01. Middle panel, cell viability assays (MTT assay) of mesoderm differentiated from hiPSCs (201B7) after 48 h of culture in the presence of 20 μM Atorvastatin and 20 μM Fluvastatin. Each measurement was performed using a microplate reader. The relative values are indicated. n = 4. Data represent the mean ± S.D. **P < 0.01. Right panel, cell viability assays (MTT assay) of endoderm differentiated from hiPSCs (201B7) after 48 h of culture in the presence of 20 μM Atorvastatin and 20 μM Fluvastatin. Each measurement was performed using a microplate reader. The relative values are indicated. n = 4. Data represent the mean ± S.D. **P < 0.01. (c) Cell viability assays (MTT assay) of myocardial cells differentiated from hiPSCs (201B7) after 48 h of culture in the presence of 20 μM Atorvastatin, Fluvastatin, Lovastatin, Mevastatin, Simvastatin and 1 μM LY294002. Each measurement was performed using a microplate reader. The relative values are indicated. n = 6. Data represent the mean ± S.D. *P < 0.05.
